# Therapeutic Interventions Targeted at Problematic Use of Digital Technology: Systematic Review and Meta-Analysis of Evidence

**DOI:** 10.2196/89280

**Published:** 2026-05-12

**Authors:** Yatan Pal Singh Balhara, Oshmita Bhattacharjee, Riya Kaur Bhatia, Rajkumar Sanahan, Ragul Ganesh, Siddharth Sarkar, Rajeev Ranjan, Shivanand Kattimani, Centre for Advanced Research on Addictive Behaviors (CAR-AB)

**Affiliations:** 1 Behavioral Addictions Clinic (BAC), Centre for Advanced Research on Addictive Behaviors (CAR-AB), ICMR Collaborative Centre of Excellence on Addictions (CCoE), All India Institute of Medical Sciences, AIIMS Ansari Nagar, New Delhi, Delhi, 110029, India, 91 1126593236; 2 National Drug Dependence Treatment Centre (NDDTC), All India Institute of Medical Sciences, New Delhi, Delhi, India; 3 Department of Psychiatry, Jawaharlal Institute of Post Graduate Medical Education and Research, Puducherry, Puducherry, India; 4 Department of Psychiatry, All India Institute of Medical Sciences, Patna, Bihar, India

**Keywords:** digital technology, internet addiction, smartphone addiction, social media addiction, gaming disorder, gambling disorder

## Abstract

**Background:**

Problematic use of digital technology has increased across the world. Despite growing research, evidence on treatment effectiveness across digital behaviors remains fragmented.

**Objective:**

This study aimed to systematically evaluate and compare the effectiveness of therapeutic interventions targeted at problematic use of digital technology across various behavioral domains.

**Methods:**

A systematic review and meta-analysis was conducted in accordance with PRISMA (Preferred Reporting Items for Systematic Reviews and Meta-Analyses) 2020 guidelines (PROSPERO: CRD420251052442). Electronic searches of PubMed, Scopus, and Embase (up to April 2025) were conducted. It identified 125 eligible studies, including 73 randomized controlled trials (RCTs), 32 non-RCTs, 14 pre-post studies, and 6 pilot studies. The interventions that were assessed in these studies included psychological therapies, digital or web-based programs, exercise-based interventions, pharmacological treatments, neuromodulation, parent-focused programs, virtual reality–based interventions, educational programs, and multicomponent approaches. Random-effects meta-analyses using standardized mean differences (SMDs) were performed.

**Results:**

For problematic internet use, psychological treatments showed a strong effect (effect size=−2.68; *P*<.001). Digital interventions also showed significant benefit (effect size=−1.16; *P*<.001). For smartphone addiction, psychological treatments (effect size=−1.49; *P*<.001) and exercise-based programs (effect size=−3.07; *P*=.001) showed significant improvement. For gaming disorder, psychological treatments showed improvement (effect size=−1.01; *P*=.02), but results were mixed. There were limited studies to calculate pooled results for social media addiction, pornography use, gambling, screen time, and over-the-top content watching. No treatment studies were found for problematic over-the-top content watching. High heterogeneity and evidence of small-study effects were observed in several studies.

**Conclusions:**

Overall, structured psychological therapies showed the most consistent benefit. These findings support structured interventions that aim for control of use and reduce cues linked to high use. Evidence remains limited for several emerging digital behaviors. More high-quality studies are needed in clinical settings and for less-studied forms of digital addiction.

## Introduction

Digital devices and the internet shape daily life. People use phones, computers, and online platforms for work, study, and leisure. These devices and tools offer ease and speed. On the flip side, constant access increases the risk of problematic use of digital technology [1]. In certain contexts, this leads to adverse consequences [2-4].

Problematic use of digital technology refers to using technology in a pattern that leads to impaired control, increasing priority given to the behavior, and continued engagement despite negative consequences. It may be associated with significant distress, dysfunction, or both. This can manifest in the form of use of digital devices (online and offline) and the internet. It can occur in various contexts including use of the internet, smartphones, gaming, social media, gambling, shopping or buying, over-the-top (OTT) content watching, pornography watching, and excessive screen time. These are all potentially addictive behaviors. While many of these have not been listed as specified diagnostic categories, *ICD-11* (*International Classification of Diseases, 11th Revision*) makes provision for listing these under the residual categories in the section on addictive behaviors [5,6].

The prevalence of problematic use of digital technology has varied widely across studies. Globally, studies place the burden of problematic use of digital technology in the general population between 6% and 15% [3]. However, the rates have been reported to be much higher among adolescents and young adults in some settings [7,8].

Various therapeutic interventions have been proposed and assessed for effectiveness in the management of problematic use of technology. These include psychological, pharmacological, educational, and neuromodulation approaches, among others. The evidence on the management of specific addictive behaviors has been compiled previously. However, previously published work has taken a piecemeal approach and failed to explore these therapeutic interventions comprehensively. For example, some of the previously published reviews have explored either one particular type of behavior [9,10] or a particular type of intervention [11,12]. Also, some of the addictive behaviors (for example, addictive behaviors associated with social media use, shopping or buying, and OTT content watching) failed to generate the same level of interest, and the evidence on the management of these remains unsystematized. More recently, there has been interest in exploring the problematic pattern of internet use [13]. However, this approach also fails to take into consideration the fact that digital technology can be used in online as well as offline mode.

Given the need for a comprehensive synthesis of evidence on interventions targeted at the management of problematic use of technology, the current systematic review and meta-analysis (SRMA) was planned. We aimed to synthesize the evidence on the treatment of problematic use of technology using a cross-cutting approach. We intended to include not only the various types of technology but also various kinds of interventions. Results will allow us to summarize the effective interventions targeted at the management of various behaviors in the context of problematic use of technology. In addition, it shall help identify gaps in the published literature that can inform the direction of future research.

## Methods

### Protocol and Registration

This SRMA followed the PRISMA (Preferred Reporting Items for Systematic Reviews and Meta-Analyses) 2020 guidelines. The protocol for SRMA was registered with the International Prospective Register of Systematic Reviews (PROSPERO) with registration ID CRD420251052442.

### Objectives

The primary objective was to evaluate the effectiveness of various interventions in managing problematic use of digital technology across domains such as internet use, smartphone use, gaming, social media use, pornography watching, gambling, OTT content watching, shopping or buying, and screen time. Secondary objectives included comparing the effectiveness of different intervention modalities; examining differential effects across age groups and conditions; and identifying methodological and quality gaps in the evidence base.

### Eligibility Criteria

#### Inclusion Criteria

Studies were included if they assessed interventions aimed at reducing or managing problematic use of digital technologies; conducted among human participants of any age; were randomized controlled trials (RCTs), non-RCTs, quasi-experimental, or pre-post intervention studies; reported quantitative outcomes on behavioral change, symptom reduction, or improvement in self-regulation related to technology use; were peer-reviewed and published in the English language.

#### Exclusion Criteria

Studies were excluded if they focused only on prevalence, correlates, or prevention of problematic use of digital technology; were nonempirical (reviews, case reports, conference proceedings, dissertations, editorials, or gray literature); focused exclusively on substance-use disorders or nondigital behaviors; lacked sufficient data to calculate effect sizes (did not report postintervention mean and SDs, change scores with variability estimates, test statistics convertible to standardized mean differences, and CIs); were published in languages other than English without an available English translation. Only interventions explicitly targeting online or digital platforms based behaviors were included. Studies focused solely on land-based or offline gambling without a digital component were excluded.

### Information Sources and Search Strategy

Electronic searches were conducted in PubMed, Scopus, and Embase for studies published up to April 2025. The search was conducted in May 2025.

Problematic use of digital technology encompassed the use of digital devices (online and offline) and the internet. It included various contexts including the use of the internet, smartphones, gaming, social media, gambling, shopping or buying, OTT content watching, pornography watching, and excessive screen time. Problematic screen time was used as a time-based construct only when it was measured independently and not embedded within a specific behavioral diagnosis. A comprehensive set of search terms combined controlled vocabulary (eg, MeSH [Medical Subject Headings]) and free-text keywords covering both the condition and intervention components. The search string included terms such as: (“excessive internet*” OR “problematic internet*” OR “internet addiction” OR “internet gaming disorder” OR “gaming disorder” OR “internet gaming disorder” OR “social media addiction” OR “pornography addiction” OR “smartphone addiction” OR “online gambling” OR “problematic online gambling*” OR “excessive screen time”) AND (“intervention*” OR “therapy” OR “treatment” OR “training” OR “program*” OR “effectiveness”).

The detailed search strings are available in Table S1 in Multimedia Appendix 1. The search targeted the title, abstract, and keyword fields. Manual searches of reference lists of relevant reviews and included studies were also conducted to identify additional articles.

### Study Selection

All search results were imported into Rayyan software for deduplication and screening. Two reviewers (RKB and RS) independently screened titles and abstracts against eligibility criteria. Full-text screening was subsequently performed by the same reviewers. Discrepancies were resolved by discussion. If needed, a senior author (YPSB) resolved the conflicts. Reasons for exclusion were documented at each stage, and the process was summarized in a PRISMA 2020 flow diagram. Additionally, an interrater reliability analysis was conducted during the initial screening process to assess the validity of the studies included in the full-text screening and data extraction.

### Data Extraction

Data were extracted independently by two reviewers (RKB and RS) using a standardized form developed in Microsoft Excel. Extracted variables included study identification (author, year, and country); design and sample characteristics (n, age, and gender distribution); intervention details (type, duration, format, and theoretical basis); comparator type (active, waitlist, or no-treatment control); outcome measures; result, effect sizes, or pre-post mean differences. Disagreements were resolved by consensus.

### Risk of Bias Assessment

The Cochrane Risk of Bias 2.0 (RoB 2) tool was used for RCTs, and the Risk Of Bias In Non-Randomized Studies - of Interventions (ROBINS-I) tool for nonrandomized and quasi-experimental studies. Each domain (randomization, allocation concealment, blinding, outcome completeness, and selective reporting) was rated as low, moderate, high, or critical risk.

### Data Synthesis and Analysis

Given anticipated heterogeneity in intervention modalities and outcome measures, both qualitative synthesis and meta-analysis were conducted. The meta-analysis was performed using Jamovi (version 2.5) [14]. Descriptive synthesis summarized intervention characteristics and results by intervention type, target behavior, and population. For studies reporting comparable quantitative data, standardized mean differences (SMDs) with 95% CIs were calculated. Random-effects models (DerSimonian–Laird) were used due to expected heterogeneity. Analyses were run for treatment-versus-control comparisons and for pre-post change within treatment arms. Statistical heterogeneity was assessed using the *I*² statistic and Cochran Q test. Subgroup analyses were pre-specified for type of intervention (psychological, digital, pharmacological, combined, etc); and type of problematic use of technology. Publication bias was evaluated using funnel plots and Egger regression test. In case an intervention was offered using a digital platform, it was categorized as a digital intervention irrespective of the nature of the intervention offered.

### Ethical Considerations

The study is based on the previously published data and did not require ethical approval.

## Results

### Study Selection and Characteristics

**Figure 1.**
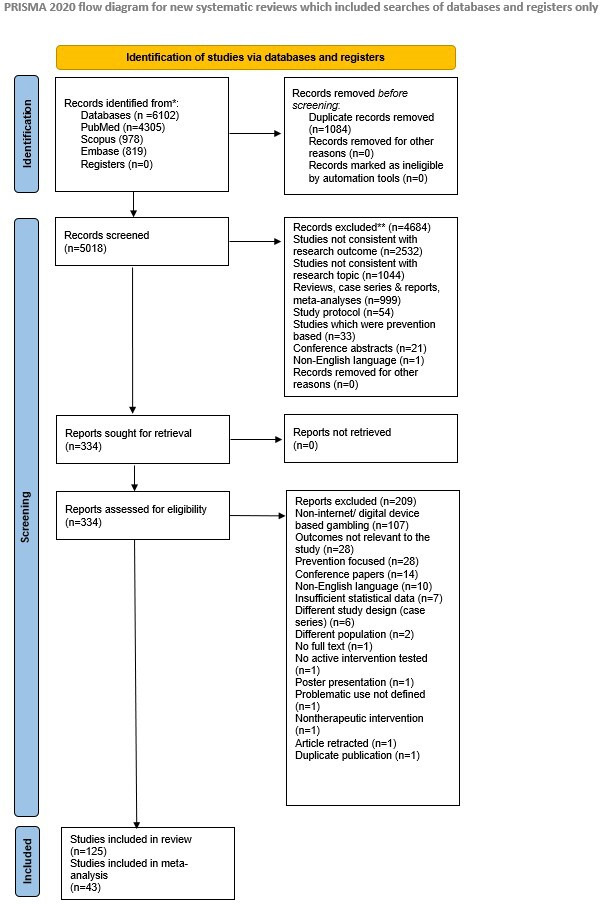
PRISMA (Preferred Reporting Items for Systematic Reviews and Meta-Analyses) flow diagram.

The initial search retrieved a total of 6102 records, using the databases of PubMed (n=4305), Scopus (n=978), and Embase (n=819). After removal of duplicates, 1206 studies remained for title and abstract screening. Overall, 125 studies [15-139] were included in the systematic review (Cohen κ value of 0.98 for interrate agreement), including RCTs (n=73) [15-17,21-24,26,31,35,37,38,40-43,47,48,50,51,54-56,58,62,63,66,67,70-72,74,75,78-81,83-85,87,91,92,95-100,106-108,111-114,116-118,120,122,124-127,131,134,135,137,139], non-RCTs (n=32) [18,33,34,36,44,45,49,52,53,59-61,64,65,76,77,88,89,93,94,101,102,105,115,119,121,123,125,130,132,133,136], pre-post single-group interventions (n=14) [28,29,32,39,57,61,69,78,86,93,101,116,138], and pilot or feasibility studies (n=6) [30,41,68,86,103,129]. Collectively, these studies included more than 8000 participants. Study publication years ranged from early 2000s to 2025(Multimedia Appendix 2) (Figure 1).

### Country Distribution

The studies were conducted across various countries including China (n=39) [16,19,25,26,30,38,41-43,45,47,48,54,62-64,66-68,70,71,73,74,83,95,98,100,103,107,114,116-118,122,123,127,129,131,139], South Korea (n=15) [46,50,58,59,65,69,85,111,120,121,125,126,133,135,140], Iran (n=11) [18,20,33,40,87,89,96,97,101,132,136], Germany (n=9) [34,36,37,91,92,99,106,137,138], United States (n=9) [39,75-77,84,93,110,113,131], Turkey (n=7) [17,51,72,88,105,108,119], India (n=6) [27,29,35,49,61,94], Australia (n=3) [52,56,81], Switzerland (n=3) [21,55,134], Indonesia (n=2) [28,90], Israel (n=2) [15,128], Sweden (n=2) [78,104], and United Kingdom (n=2) [31,79]. One study each was published from Bosnia and Herzegovina, Brazil, Egypt, Finland, France, Greece, Indonesia, Malaysia, Japan, Latvia, Nigeria, Norway, the Philippines, Spain, and Thailand.

### Study Settings

The interventions were implemented in diverse settings including schools (n=31) [15-17,20,26-29,33,48-51,65-70,82,85,88,89,96,105,109,115,123,126,132,133], colleges or universities (n=36) [18,19,22-24,30,34,38,40,47,53,58,66,68,70,72,73,75,76,83,90,92,96,100,102,112,116,117,119,125,127,130,131,136], hospitals or clinical settings (n=31) [34-37,43,44,46,54-56,60-64,77,78,81,92,93,102,110,111,113,114,118,120-122,124,125,135], digital platforms (n=17) [56,81,84,91,94,95,97,99-101,103,106-108,113,137,138], laboratory or field (n=3) [75,122,134], and community settings (n=5) [70,71,74,118,123]. Participants included adolescents, university students, and adults aged between 12 and 50 years, with sample sizes ranging from 6 to above 800 participants and intervention durations of 2 to 24 weeks.

### Targeted Problematic Use of Technology

Across all intervention categories, various behaviors in the context of problematic use of technology were targeted including internet addiction (IA) or problematic internet use (PIU) or internet use disorders (IUD; n=52) [15-43,75-77,91-102,110,114,115,123,128,131,136-138], internet gaming disorder or problematic gaming or problematic online gaming or gaming disorder (n=35) [44-64,85-87,103-105,111,112,120-122,125,130,131], smartphone addiction or problematic smartphone use or mobile phone dependence (n=23) [65-74,83,88-90,106,107,116-118,126,127], problematic gambling or gambling disorder (n=5) [78-81,113], problematic screen time or excessive screen time (n=4) [84,109,119,129], social media addiction or problematic social media use (n=3) [82,83,108], and problematic pornography watching (n=3) [75-77].

### Psychological Interventions

#### Overview

Seventy-five studies [15-84] assessed psychological and behavioral interventions aimed at reducing problematic technology use across seven conditions. These included structured Cognitive Behavioral Therapy (CBT), Dialectical Behavior Therapy (DBT), Acceptance and Commitment Therapy (ACT), Mindfulness-Based Cognitive Therapy (MBCT), Solution-Focused Therapy (SFT), metacognitive practices, emotion-regulation training, motivational methods, and behavioral modification techniques such as self-monitoring, cognitive bias training, and relapse prevention. Most trials involved adolescents or university students and were delivered in schools, colleges, clinics, or online environments. Intervention length ranged from 2 to 12 weeks. Outcomes were measured using validated scales, including the Internet Addiction Test (IAT), Internet Gaming Disorder Scale-Short Form (IGDS9-SF), Smartphone Addiction Scale-Short Version (SAS-SV), and Problem Gambling Severity Index (PGSI).

#### IA or PIU or IUDs

Thirty-one studies [15-43] examined psychological and behavioral treatments for IA or PIU or IUDs. School-based CBT programs reduced IA scores and improved self-control in adolescents [15,16]. Solution-focused group work with parental psychoeducation produced large decreases in PIU [17]. CBT with cognitive restructuring and relapse prevention reduced IA in young adults and improved quality of life [18]. Brief CBT groups also showed early gains in coping and self-regulation [19]. CBT-based emotion-regulation tailored to behavioral inhibition system or behavioral activation system (BIS/BAS) profiles also reduced IA [20]. Online CBT and motivational interviewing improved problematic pornography use [21]. Online CBT sessions improved self-monitoring and self-regulation and reduced IA in young adults [22]. Parent-child interaction sessions reduced IA and improved sleep and self-efficacy [22]. CBT-based school programs produced improvements in time management and emotional control [16]. After-school CBT groups showed large reductions in PIU that were sustained at follow-up [23]. Distress-tolerance training reduced PIU and improved well-being [24]. Multifamily group therapy sharply reduced IA prevalence and improved family relationships [25]. Logotherapy-based mindfulness lowered IA scores and improved coping [26]. School-based interventions with parent sessions improved PIU and functioning [27]. Reality-therapy group counseling reduced PIU in adolescents [28]. Short psychoeducation programs demonstrated steady reductions at follow-up [29]. Solution-focused brief therapy reduced Chen Internet Addiction Scale - Revised (CIAS-R) scores and improved future time orientation [30]. App-based mindfulness outperformed relaxation training [31]. CBT psychoeducation improved coping, reduced cravings, and lowered symptoms in a mixed PIU sample [32]. MBCT reduced IA and improved general health [33]. PROTECT, a CBT-based program, reduced IUD symptoms and emotional problems over a 12-month follow-up [34]. Combined psychotherapy and yoga reduced internet and smartphone use more than psychotherapy alone [35]. STICA, a structured CBT program, reduced IA severity in both single-arm and multicenter RCT designs [36,37]. Weekend group psychoeducation improved IA, sense of coherence, and social support [38]. CBT-IA reduced IA symptoms and maintained gains at 1, 3, and 6-month follow-ups [39]. Group CBT reduced compulsive use and Young’s Internet Addiction Test (YIAT) scores with strong effects [40]. Group counseling reduced CIAS-R and improved mental health, with gains maintained at 6 months [41]. Psychosocial mutual-help groups reduced IA more than standard withdrawal [42]. Family-based CBT reduced IA more than control and improved family functioning [43].

#### Internet Gaming Disorder or Problematic Gaming or Problematic Online Gaming or Gaming Disorder

Overall, 22 studies [44-64,131] assessed psychological interventions for internet gaming disorder (IGD) or problematic gaming or problematic online gaming or gaming disorder. A multicomponent CBT program (PIPATIC) reduced IGD symptoms more than control over 6 months [44]. Mindfulness-based craving-control training reduced symptoms and maintained gains at follow-up [45]. Group CBT outperformed supportive therapy in reducing IGD severity [46]. Cognitive bias modification reduced craving and attentional bias [47]. An 8-week online internet-based CBT program reduced gaming time, motivations, and symptom severity [48]. A brief school program with youth and parent sessions showed slight nonsignificant improvement [49]. Art-therapy and exercise camps both reduced symptoms with no between-group differences [50]. A 4-month solution-focused program reduced IAT and sleep-related problems, with effects sustained at 6 months [51]. Short-term abstinence with daily monitoring produced meaningful improvement for most participants [52]. A mindfulness-based cognitive-restructuring program reduced IGD and improved well-being [53]. Mindfulness-meditation reduced IGD symptoms and cravings more than progressive muscle relaxation [54]. Multidimensional Family Therapy produced greater reductions than treatment as usual at 6- and 12-month follow-ups [55]. A large trial found small improvements in all groups, with relaxation slightly outperforming control and mindfulness showing no clear advantage [56]. An eclectic CBT-based program showed moderate clinician-rated improvements [57]. CBT reduced IGD more than virtual-reality therapy [58]. Equine-assisted learning reduced IGD with partial relapse at follow-up [59]. A short residential program combining CBT, psychoeducation, and counseling reduced gaming time and improved motivation [60]. A ten-session motivational-CBT intervention reduced IGD symptoms and improved quality of life [61]. Approach-bias training reduced IGD severity and craving, while sham training showed no change [62]. Mindfulness reduced craving and IGD more than progressive muscle relaxation in an 8-session program [63]. A craving-behavioral intervention with mindfulness reduced CIAS scores and craving more than control [64].

#### Smartphone Addiction or Problematic Smartphone Use or Mobile Phone Dependence

Twelve psychological studies [65-74,134,135] targeted smartphone addiction or problematic smartphone use or mobile phone dependence. School-based mindfulness programs reduced addiction scores and improved self-control and emotion regulation [65,66]. An 8-week emotional-processing program outperformed CBT and waitlist control, with sustained reductions in problematic use [67]. Combined CBT and mindfulness reduced addiction and phone-use time in young adults, although gains weakened at longer follow-up [68]. A short CBT diary–based program with parent involvement reduced addiction in early adolescents [69]. Brief body-scan mindfulness sessions lowered phone addiction and increased mindfulness and meaning in life [70]. Mindfulness education integrating attention control, emotional regulation, and time management produced large reductions in mobile phone addiction index scores [71]. Online psychoeducation covering time management, emotional regulation, and coping produced sustained reductions over 6 months [72]. MBCT combined with metaphor therapy reduced problematic social media use and maintained gains at follow-up [73]. A 12-week health action process approach-based counseling program reduced mobile phone addiction and increased self-efficacy [74].

#### Problematic Pornography Watching

Three studies [75-77] evaluated psychological treatment for problematic pornography watching. ACT-based programs showed the strongest effects. A 12-week ACT intervention produced large reductions in pornography use, with most participants achieving near-cessation and maintaining gains at follow-up [75]. An earlier ACT trial reported similar reductions in viewing time and improved psychological flexibility [76]. A multicomponent group program that combined CBT, psychodynamic work, and motivational interviewing improved mood and depressive symptoms but did not significantly reduce problematic sexual behavior [77].

#### Problematic Gambling or Gambling Disorder

Four studies [78-81,113] evaluated psychological approaches for problematic gambling or gambling disorder related specifically to online or digital gambling platforms. CBT-based work reduced symptoms of digital gambling in adolescents, although changes in broader problem-gambling scores were limited [78]. A large trial of online responsible-gambling messages found small reductions in gambling behavior across all groups, with no clear advantage for tailored content [79]. A web-based program combining normative feedback and CBT modules produced reductions in gambling severity and frequency, but effects did not differ across delivery formats [80]. Cognitive therapy and exposure therapy both reduced gambling-related beliefs and distress, with similar improvements across treatment conditions [81].

#### Social Media Addiction or Problematic Social Media Use

Two studies [82,83] assessed psychological interventions for social media addiction or problematic social media use. A school-based CBT program that included media literacy, time management, and self-control training reduced addiction scores and daily use among adolescents, with gains maintained at follow-up [82]. A mindfulness-based program that integrated formal and informal practice reduced social media addiction or problematic social media use and improved stress and mood outcomes in young adults [83].

#### Problematic Screen Time or Excessive Screen Time

One study [84] targeted problematic screen time or excessive screen time. A 3-week CBT-based program that combined cognitive restructuring, coping-skills training, and relapse prevention reduced daily screen use and increased physical activity, with gains sustained across a 20-week follow-up [84].

### Educational Interventions

Six studies [85-90] tested educational programs that aimed to raise awareness, teach self-regulation, and promote healthier digital habits. Interventions were school- or university-based and ranged from a single-day workshop to multiweek counseling with follow-up messages. Outcome measures included the Smartphone Addiction Scale for Adolescents (SASA), IAT, Young Internet Addiction Scale (YIAS), and measures of readiness to change and self-efficacy.

#### Internet Gaming Disorder or Problematic Gaming or Problematic Online Gaming or Gaming Disorder

Three studies [85-87] targeted internet gaming disorder or problematic gaming or problematic online gaming or gaming disorder. Chung et al [85] compared two 4-week, eight-session school-based courses—game coding and gaming-literacy training—and found significant reductions in YIAS scores in both groups, with larger improvements in the coding arm [85]. Männikkö et al [86] delivered a 10-session psychoeducational curriculum focused on gaming awareness and self-regulation; effects on gaming time and severity were small and nonsignificant. Zamanian et al [87] implemented an 8-session Theory of Planned Behavior–based program with an added parent workshop and reported a modest but significant reduction in gaming-dependency scores post intervention, with a small rebound at follow-up [87].

#### Smartphone Addiction or Problematic Smartphone Use or Mobile Phone Dependence

Three studies [88-90] targeted smartphone addiction or problematic smartphone use or mobile phone dependence. Ertemel and Ari [88] implemented a 1-day, 8-module school program covering digital habits, screen tracking, and healthier device use, and reported significant reductions in SASA scores. Noroozi et al [89] delivered an 8-week transtheoretical-model program with counseling, weekly motivational messages, and self-monitoring tasks and observed clear reductions in mobile phone addiction and increased readiness for change. Shen and Rukmini [90] compared printed leaflets with infographic delivery over one month and found larger reductions in SAS scores in the printed-materials group.

### Digital Interventions

Twenty-two studies [91-109,136-138] evaluated digital or technology-delivered interventions, including web-based CBT, online ACT, virtual reality (VR) modules, telehealth psychotherapy, online meditation, photography-based reflection tasks, digital education, and app-based mindfulness or self-monitoring tools. Program duration ranged from 10 days to 12 weeks. Most interventions used asynchronous modules, telehealth guidance, or app-based practice tasks.

#### IA or PIU or IUDs

Fifteen studies [91-102,136-138] targeted IA or PIU or IUDs. Web-based CBT and telehealth psychotherapy reduced symptom severity and improved coping across multiple trials [91-93]. Online meditation and mindfulness programs produced significant reductions in YIAT scores and improved quality of life [94]. Photography-and-reflection tasks reduced IAT scores and improved mood and perceived control [95]. Working-memory training delivered through adaptive electronic working memory training modules improved cognitive control and reduced PIU severity, with gains sustained at follow-up [96,97]. Cognitive bias modification targeting approach tendencies reduced craving and gaming-related reactivity [98]. Motivational programs improved readiness to change and reduced symptoms of media addiction [99]. A system-based CBT module (HOSC) reduced online hours and Young’s Diagnostic Questionnaire (YDQ) scores across active-training arms [100]. Integrative online emotion-regulation training reduced IA severity and improved coping strategies, with maintained follow-up gains [101]. Online DBT and CBT programs reduced symptoms of internet addiction; there was no difference between groups [102].

#### Internet Gaming Disorder or Problematic Gaming or Problematic Online Gaming or Gaming Disorder

Three studies [103-105] targeted internet gaming disorder or problematic gaming or problematic online gaming or gaming disorder. Two self-help ACT programs delivered online reduced gaming severity and weekly gaming frequency; the engaged ACT format produced larger reductions in daily gaming hours and greater gains in psychological flexibility [103]. Relapse-prevention CBT delivered in person or via video improved IGD symptoms more than treatment-as-usual, with reductions maintained at follow-up [104]. A school-based digital activity program that incorporated structured traditional games reduced internet use frequency in children over an 8-week period [105].

Another study included participants with disordered gaming [78]. However, these participants were also included in the final publication based on this study and were reported as part of that study [104]. The results for disordered gaming for the pilot study have not been presented separately to avoid duplication.

#### Smartphone Addiction or Problematic Smartphone Use or Mobile Phone Dependence

Two studies [106,107] examined interventions for smartphone addiction or problematic smartphone use or mobile phone dependence. A 20-day app-based program combining mindfulness and impulse-control modules reduced problematic use and daily screen time, although similar reductions were also seen in the control condition [106]. A 30-day online mindfulness protocol that used brief daily practice produced clear reductions across smartphone-addiction subscales and increased mindfulness [107].

#### Social Media Addiction or Problematic Social Media Use

One study [108] tested a 12-session online Roy Adaptation Model–based program to reduce social media addiction or problematic social media use. A 12-session online Roy Adaptation Model–based program improved healthy lifestyle behaviors and reflective thinking but did not reduce social media addiction or problematic social media use scores [108].

#### Problematic Screen Time or Excessive Screen Time

One digital program targeted excessive screen time in children. A 4-week mother-focused intervention delivered through YouTube animations and WhatsApp guidance reduced children’s daily screen time and improved parental monitoring and rule consistency, with effects maintained at follow-up [109].

### Pharmacological Interventions

Four studies evaluated pharmacological agents for internet, gaming, and gambling-related problems. These trials tested antidepressants (bupropion and escitalopram), antipsychotics (olanzapine), stimulants (methylphenidate), and noradrenergic agents (atomoxetine). Treatment duration ranged from 7 to 12 weeks. Outcomes were assessed using the YIAS, Clinical Global Impression – Improvement Scale (CGI-I), Bergen Internet Addiction Scale (BGCS), PGSI-related scales, and psychiatric symptom measures.

#### IA or PIU or IUDs

One study targeted impulsive-compulsive internet use. A 10-week open-label escitalopram trial reduced weekly nonessential internet use and improved global functioning, but a subsequent 9-week placebo-controlled phase showed no significant difference between escitalopram and placebo [110].

#### Internet Gaming Disorder or Problematic Gaming or Problematic Online Gaming or Gaming Disorder

Two studies [111,112] evaluated pharmacological treatment for internet gaming disorder or problematic gaming or problematic online gaming or gaming disorder.

Bupropion and escitalopram, combined with psychoeducation, reduced IGD symptoms over a 12-week period, with bupropion showing numerically larger improvements, although group differences were not significant [111]. A separate trial of methylphenidate and atomoxetine found no significant reductions in gaming severity or impulsivity, although methylphenidate showed some advantage at follow-up [112].

#### Problematic Gambling or Gambling Disorder

One study assessed pharmacological treatment for problematic gambling. A 7-week placebo-controlled trial of olanzapine reduced gambling urges in both groups, with no clear advantage for the active medication; gambling time decreased, but craving levels were higher in the treatment arm [113].

### Physical or Exercise-Based Interventions

Seven studies evaluated exercise-based interventions aimed at reducing problematic technology use. Programs included aerobic exercise, tai chi, Qigong, structured physical activity, treadmill training, and recreational sports. Intervention lengths ranged from 8 to 15 weeks, with sessions delivered 2 to 3 times per week.

#### IA or PIU or IUDs

Two studies [114,115] tested exercise programs for internet addiction or problematic internet use or internet use disorders. An 8-week exercise program significantly reduced IAT scores compared to a control group, while tai chi showed no significant effect [114]. A 15-week school- and home-based physical activity program found no meaningful changes in problematic internet use in either structured (FSPEP - Fully Supervised Physical Exercise Program) or flexible (PSPEP - Partly Supervised Physical Exercise Program) formats [115].

#### Smartphone Addiction or Problematic Smartphone Use or Mobile Phone Dependence

Four studies [38,116-118] examined physical activity to reduce smartphone addiction or problematic smartphone use or mobile phone dependence. A 12-week ME-Qigong program reduced problematic use more than CBT and control groups, with the largest improvements seen in the Qigong arm [116]. Structured activity sessions using Baduanjin or basketball also produced significant reductions in smartphone addiction compared to control [117]. Aerobic treadmill sessions led to a moderate reduction in mobile phone craving relative to a music-listening control [38]. An 8-week community program found reductions across aerobic and tai chi groups, with no meaningful differences between activity types [118].

#### Problematic Screen Time or Excessive Screen Time

One study targeted excessive screen time. A multisport recreational program conducted three times per week produced significant reductions in daily screen time, while the control group showed a slight increase [119].

### Neuromodulation-Based Interventions

#### Overview

Three studies evaluated neuromodulation for problematic technology use. All used transcranial direct-current stimulation (tDCS) targeting the dorsolateral prefrontal cortex (DLPFC), delivered across 10‐12 sessions over four weeks. Outcomes included IAT, craving indices, inhibitory-control measures, and weekly gaming hours. Neuroimaging outcomes were reported in two studies.

#### Internet Gaming Disorder or Problematic Gaming or Problematic Online Gaming or Gaming Disorder

Three tDCS studies [120-122] targeted internet gaming disorder or problematic gaming or problematic online gaming or gaming disorder. A 4-week active tDCS protocol reduced weekly gaming hours and improved self-control compared with sham stimulation [120]. A similar 12-session tDCS program targeting the bilateral DLPFC reduced IAT scores and gaming time in a pre-post design [121]. A crossover study found that active right-DLPFC stimulation reduced background craving, while sham stimulation showed no effect [122].

### Combined or Multicomponent Based Interventions

Five studies evaluated multicomponent interventions integrating psychological, behavioral, and physical elements. These programs combined modalities such as CBT, narrative counseling, psychoeducation, mindfulness, and structured exercise. Interventions ranged from 5 to 10 sessions delivered over 6‐8 weeks and targeted multiple mechanisms, including impulsivity, emotional regulation, craving, attentional control, and functional impairment.

#### IA or PIU or IUDs

Two studies [123,124] used combined interventions for IA or PIU or IUDs. An 8-week dual-modality program that integrated narrative counseling with Pilates exercises produced significant reductions in IA symptoms compared with controls [123]. A trial comparing electroacupuncture, CBT-based psychological intervention, and a combined program found reductions across all groups, with the combined treatment producing the largest improvement in IA scores [124].

#### Internet Gaming Disorder or Problematic Gaming or Problematic Online Gaming or Gaming Disorder

One multimodal trial targeted internet gaming disorder. Han et al [125] compared bupropion SR (150 to 300 mg/day) with placebo alongside weekly psychoeducation covering healthy internet use, addiction mechanisms, and coping strategies. Significant reductions were observed in YIAS scores (71.5 to 45.2 vs 68.5 to 59.2) and weekly gaming hours (47.3 to 21.1 vs 44.3 to 29.8). Improvements were maintained at 4-week follow-up.

#### Smartphone Addiction or Problematic Smartphone Use or Mobile Phone Dependence

Two studies [126,127] tested combined approaches for smartphone addiction or problematic smartphone use or mobile phone dependence. An 8-week program integrating weekly CBT-based group work with structured music-therapy sessions reduced smartphone and internet addiction scores and improved emotional symptoms in adolescents [126]. A mindfulness-based Tai Chi Chuan program that combined movement practice, breathing work, and brief meditation reduced mobile phone addiction and improved mindfulness and executive function in young adult males [127].

### Interventions With Parents

Two studies assessed parent-focused interventions aimed at reducing problematic internet use and excessive screen time in children. These programs targeted parental behavior, family communication, and home-based digital regulation practices. Interventions ranged from structured parent-training sessions to multicomponent community programs delivered in a cluster-randomized design.

#### IA or PIU or IUDs

One study evaluated a parent-directed program for problematic internet use. A 3-session parent vigilant care parent-training program that taught active and restrictive mediation strategies reduced child PIU scores and improved parent–child functioning, with gains maintained at follow-up [128].

#### Problematic Screen Time or Excessive Screen Time

One study targeted home-based screen-time reduction in children using a cluster-randomized, waitlist controlled design. A cluster-randomized trial found that the intervention reduced daily and weekly screen time and lowered entertainment-use duration compared with a waitlist control, although it did not change parenting practices or physical activity levels [129].

### VR-Based Interventions

#### Overview

One study evaluated VR-based treatment for problematic digital behavior. The intervention used immersive VR to support mindfulness, attentional control, and emotional regulation. The program was delivered through a VR health platform with weekly sessions over 4 weeks.

#### Internet Gaming Disorder or Problematic Gaming or Problematic Online Gaming or Gaming Disorder

One study assessed a meditation-based intervention for internet gaming disorder or problematic gaming or problematic online gaming or gaming disorder. A 4-week program using brief weekly mindfulness sessions delivered through an extended reality (XR) platform reduced IGD symptoms and lowered weekend gaming time, with no significant change in weekday use [130].

There were no interventions targeting problematic OTT content watching.

### Meta-Analysis Results

#### Overview

Random-effects models were used to synthesize results across intervention types and target behaviors. Analyses were run for treatment-versus-control comparisons and for pre-post change within treatment arms. Heterogeneity was assessed with *I*², Q, and τ². Publication bias was evaluated using Egger regression, Begg–Mazumdar rank correlation, and fail-safe N. All models used the DerSimonian–Laird estimator.

#### IA or PIU or IUDs

##### Psychological Interventions

**Figure 2.**
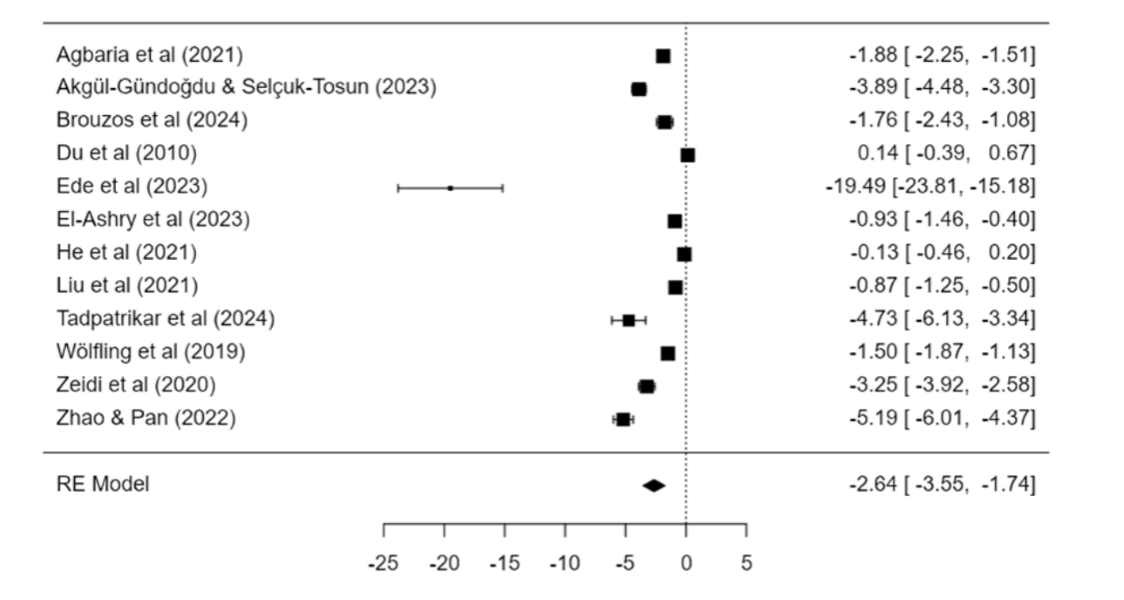
Forest plot displaying the individual study effect sizes and the pooled overall effect derived from a random-effects model for psychological interventions for internet addiction or problematic internet use or internet use disorders [15-17,22-24,26,35,37,40,42,47].

Psychological treatments produced a clear reduction in symptoms compared with control conditions (estimate=−2.68, SE 0.45, *P*<.001; 95% CI −3.57 to −1.80). Heterogeneity was high (*I*²=96.47%, Q(11)=311.97, *P*<.001, τ²=2.18). Pre-post effects were also significant (estimate=4.64, SE 1.86, *P*=.01; 95% CI 1 to 8.28), with marked heterogeneity (*I*²=98.88%). Egger regression suggested small-study effects (*P*<.001). Trim-and-fill did not impute missing studies (Figure 2).

##### Digital Interventions

**Figure 3.**
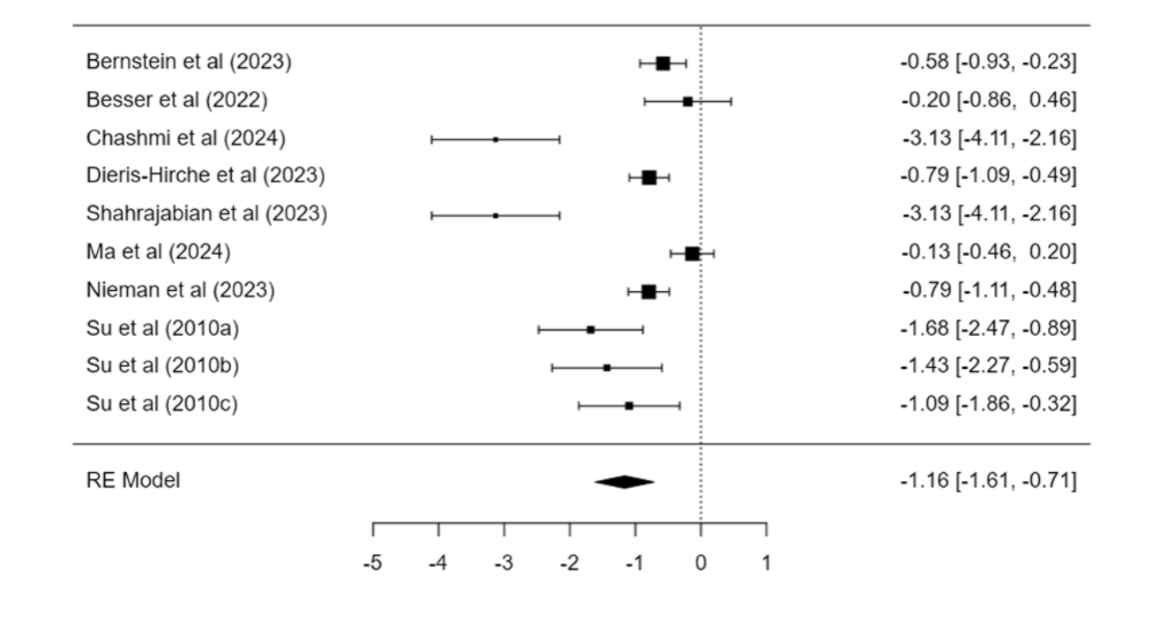
Forest plot displaying the individual study effect sizes and the pooled overall effect derived from a random-effects model for digital interventions for internet addiction or problematic internet use or internet use disorders [91,92,95-97,99,100,137].

Digital-based treatment showed a significant pooled effect relative to controls (estimate=−1.16, SE 0.23, *P*<.001; 95% CI −1.61 to −0.72). Heterogeneity was high (*I*²=87.48%, Q(9)=71.90, *P*<.001). Pre-post change was also significant (estimate=0.85, SE 0.20, *P*<.001), and showed no heterogeneity (*I*²=0%). Egger test was significant (*P*<.001). Trim-and-fill did not alter results (Figure 3).

##### Exercise-Based Interventions, Educational, Neuromodulation, and Combined Interventions

Evidence within these groups was limited and did not support pooled estimates.

### Internet Gaming Disorder or Problematic Gaming or Problematic Online Gaming or Gaming Disorder

**Figure 4.**
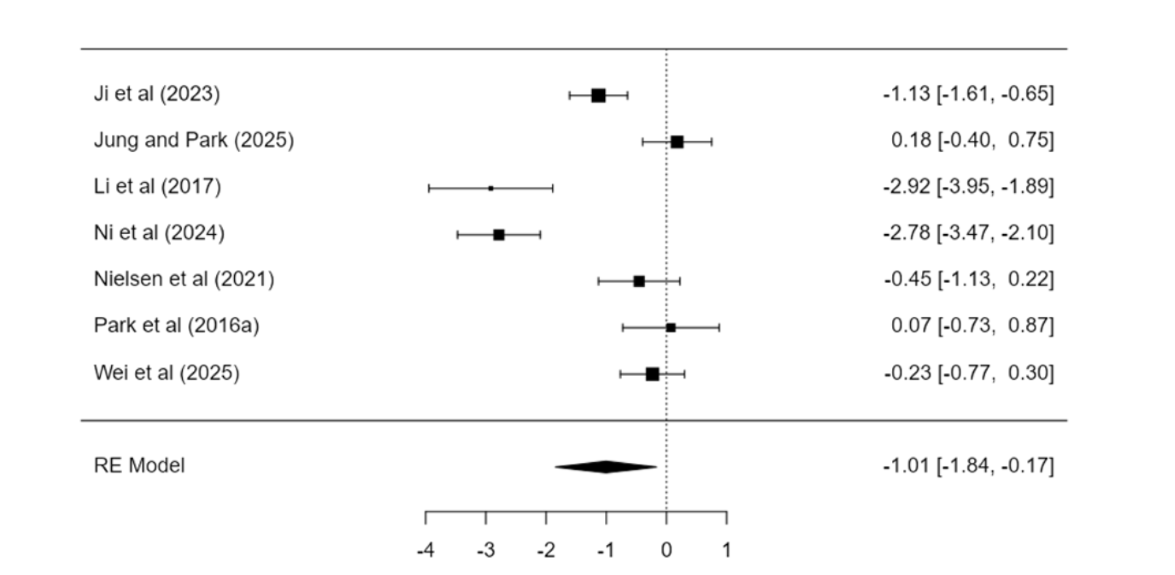
Forest plot displaying the individual study effect sizes and the pooled overall effect derived from a random-effects model for psychological interventions for internet gaming disorder or problematic gaming or problematic online gaming or gaming disorder [48,50,54,55,58,62,131].

Treatment–control differences were significant (estimate= −1.01, SE 0.42, *P*=.02). Heterogeneity was high (*I*²=91.57%). Pre-post change did not meet significance (estimate=2.51, SE 1.41, *P*=.07; 95% CI −0.24 to 5.27), with marked heterogeneity (*I*²=96.36%; Figure 4).

### Digital Interventions, Exercise-Based Interventions, Educational, Neuromodulation, and Combined Interventions

Evidence within these groups was limited and did not support pooled estimates.

### Smartphone Addiction or Problematic Smartphone Use or Mobile Phone Dependence

#### Psychological Interventions

**Figure 5.**
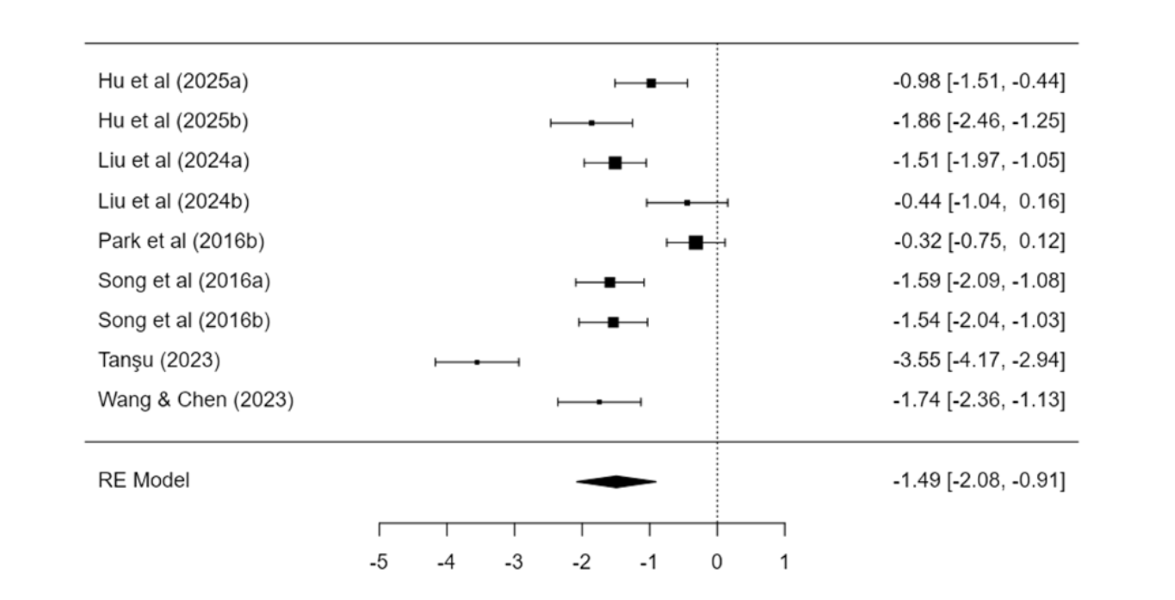
Forest plot displaying the individual study effect sizes and the pooled overall effect derived from a random-effects model for psychological interventions for smartphone addiction or problematic smartphone use or mobile phone dependence [58,66,67,71,72,135].

Psychological treatment led to a clear drop in symptoms versus control (estimate=−1.49, SE 0.30, *P*<.001; 95% CI −2.08 to −0.90). Heterogeneity was high *(I*²=90.88%). Pre–post change was significant (estimate=2.08, SE 0.39, *P*<.001) with no heterogeneity (*I*²=0%). Egger test did not show strong bias (*P*=.11), and no missing studies were imputed (Figure 5).

#### Exercise-Based Interventions

**Figure 6.**
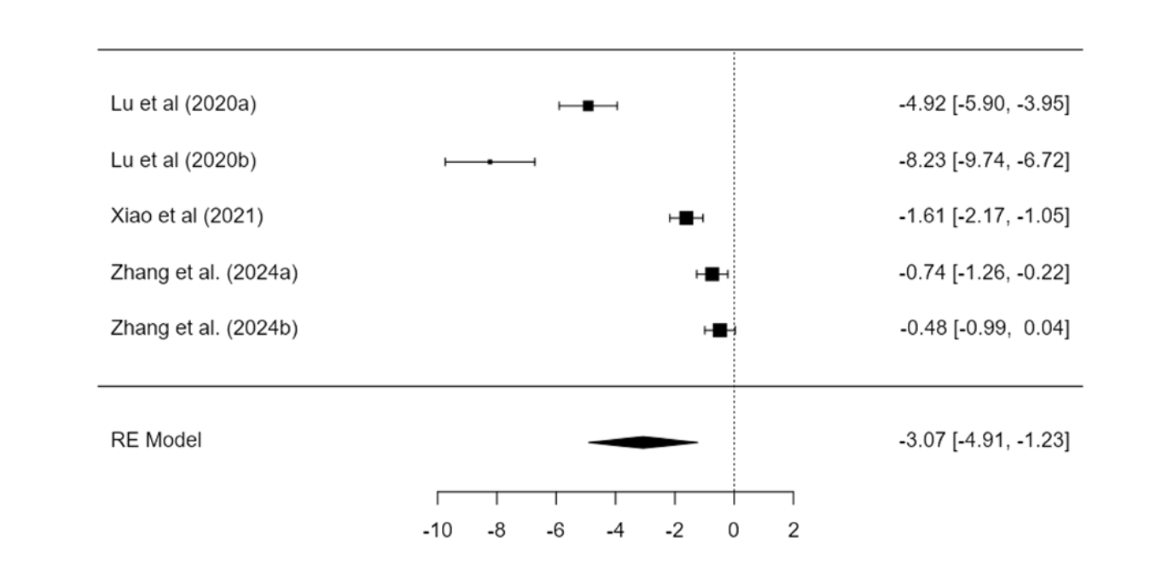
Forest plot displaying the individual study effect sizes and the pooled overall effect derived from a random-effects model for exercise-based interventions for smartphone addiction or problematic smartphone use or mobile phone dependence [116-118].

Treatment–control effects were significant (estimate=−3.07, SE 0.94, *P*=.001). Heterogeneity was high (*I*²=97.28%). Pre–post effects were significant (estimate=4.17, SE 1.52, *P*=.006; Figure 6).

#### Digital Interventions, Educational, Neuromodulation, and Combined Interventions

Evidence within these groups was limited and did not support pooled estimates.

No pooled results were possible for problematic pornography watching, problematic gambling or gambling disorder, social media addiction or problematic social media use, or problematic screen time or excessive screen time due to insufficient trial data.

### Risk of Bias Assessment

**Figure 7.**
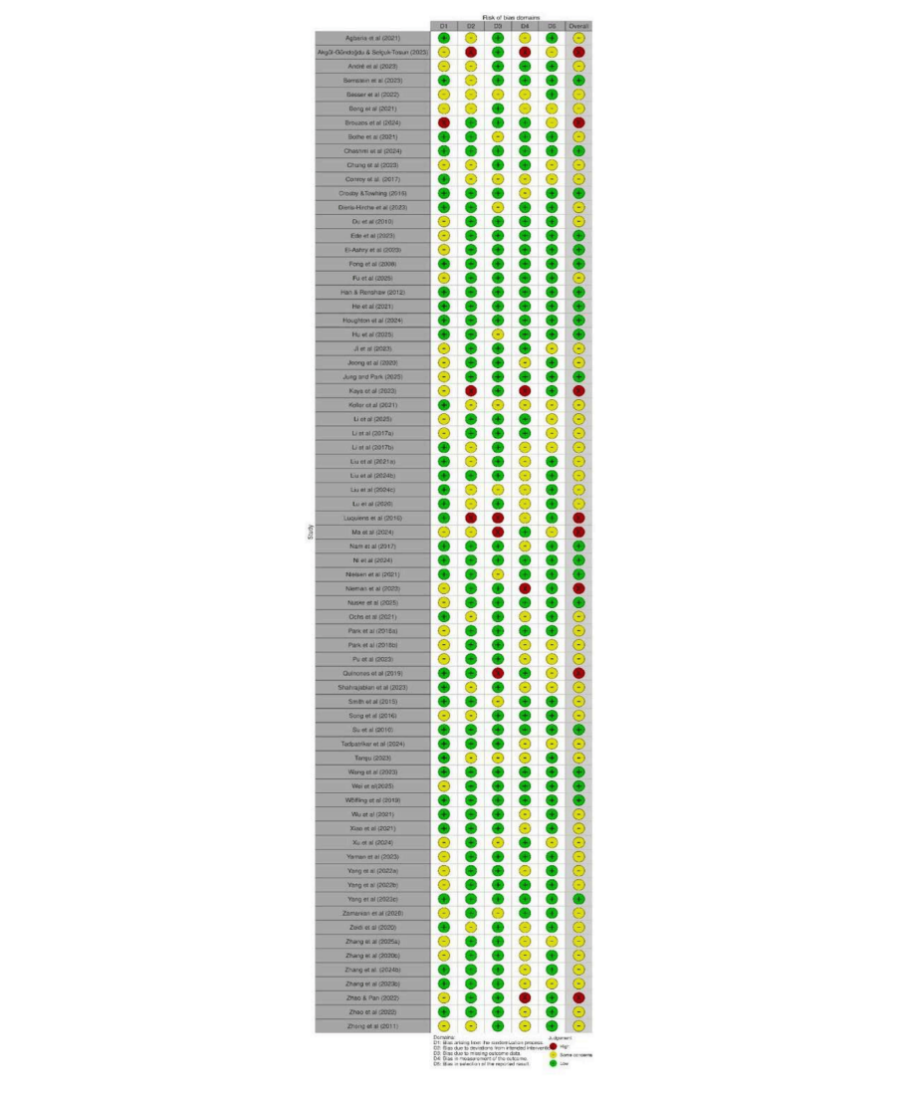
Traffic light figure for randomized controlled trial studies [15,17,21-24,26,30,31,35,37,38,40-43,47,48,51,54-56,58,62,63,66,71,72,74,75,79-81,84,85,87,91,92,95-100,104,106-108,111-114,116-118,120,122,124-127,131,134,135,137,139].

**Figure 8.**
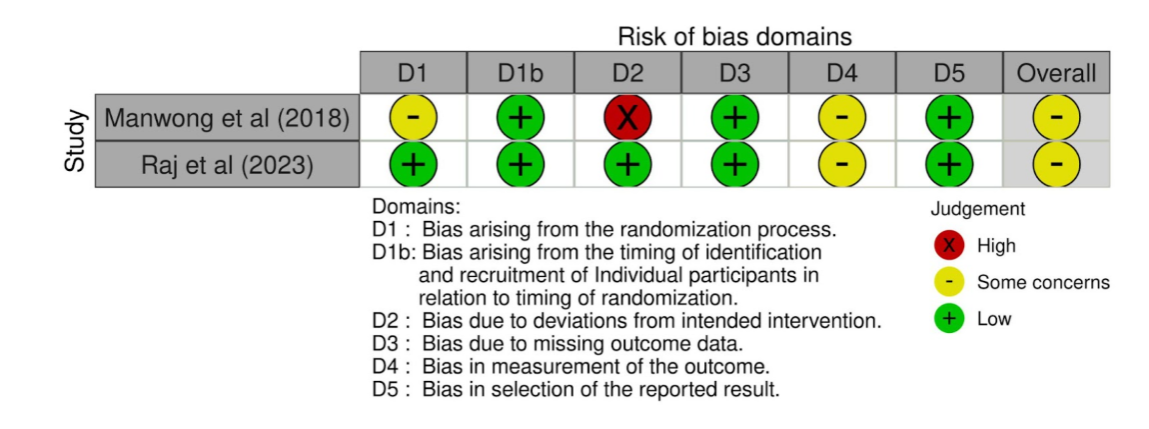
Traffic light figure for cluster randomized studies [82,109].

**Figure 9.**
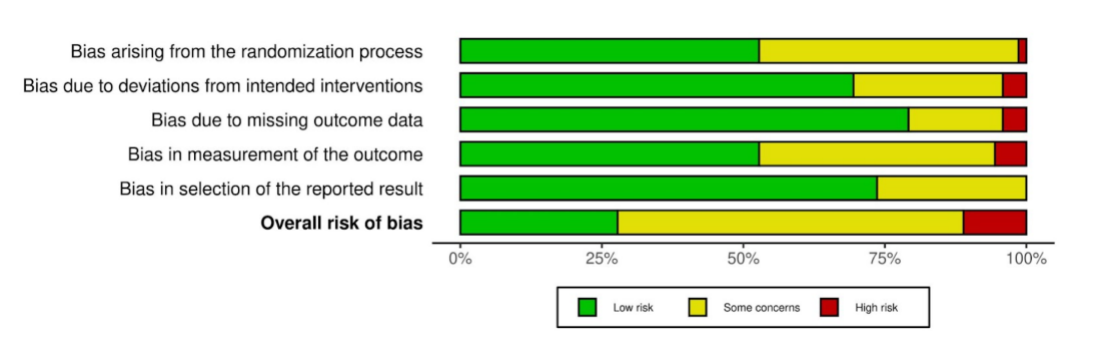
Weighted bar plots of the distribution of risk-of-bias judgements within each bias domain for randomized studies.

The overall methodological quality of the included studies varied considerably across intervention categories. Based on Cochrane RoB 2 (for RCTs) criteria, 44 (61.9%) studies [15,21,26,30,35,38,40-43,48,58,63,66,72,74,75,81,84,85,87,92,97,98,104,106-108,112,114,116-118,120,122,124,126,127,131,134,135,137,139] were rated as having some concern, 19 (26.7%) studies [23,24,37,38,47,54-56,62,71,75,79,91,96,100,111,113,125] as low risk, and 8 (11.2 %) [17,22,31,42,51,80,95,99] as high risk of bias. Most RCTs demonstrated generally low risk of bias across the majority of domains (Figure 7). Bias arising from the randomization process, bias due to missing outcome data, and bias in measurement of the outcome were predominantly rated as low risk, indicating that random allocation, retention, and outcome assessment procedures were typically well controlled. However, a notable proportion of studies showed “some concerns” in the domains related to deviations from intended interventions and selection of the reported result, suggesting incomplete reporting of adherence, protocol deviations, or prespecified analysis plans. Only a small number of studies were judged to have a high overall risk of bias, mostly due to issues with outcome reporting or insufficient methodological transparency (Figure 8). Overall, the evidence base from randomized trials can be considered methodologically robust, although interpretation should account for selective reporting and incomplete intervention fidelity in some studies (Figure 9).

The 2 RCTs [82,109] showed predominantly low risk of bias across most assessed domains. Both trials demonstrated low risk in the randomization process, missing outcome data, and outcome measurement, indicating appropriate allocation procedures and reliable outcome reporting. However, Manwong et al [82] exhibited high risk in deviations from the intended intervention, likely due to insufficient adherence reporting or uncontrolled protocol deviations. Additionally, both studies showed “some concerns” in the timing of participant recruitment and in the selection of reported results, suggesting potential issues with prespecification or full disclosure of outcomes. Overall, both trials were rated as having some concerns, reflecting generally sound methodology but with minor limitations that should be considered in interpretation of results.

**Figure 10.**
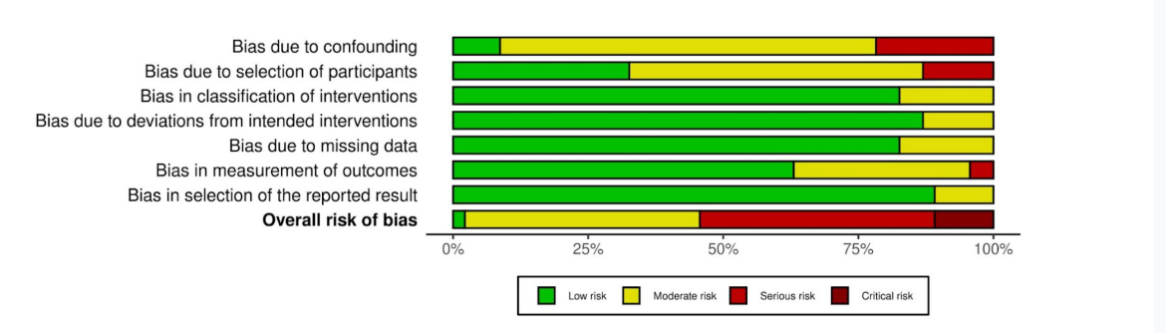
Weighted bar plots of the distribution of risk-of-bias judgements within each bias domain for nonrandomized and quasi-experimental studies.

**Figure 11.**
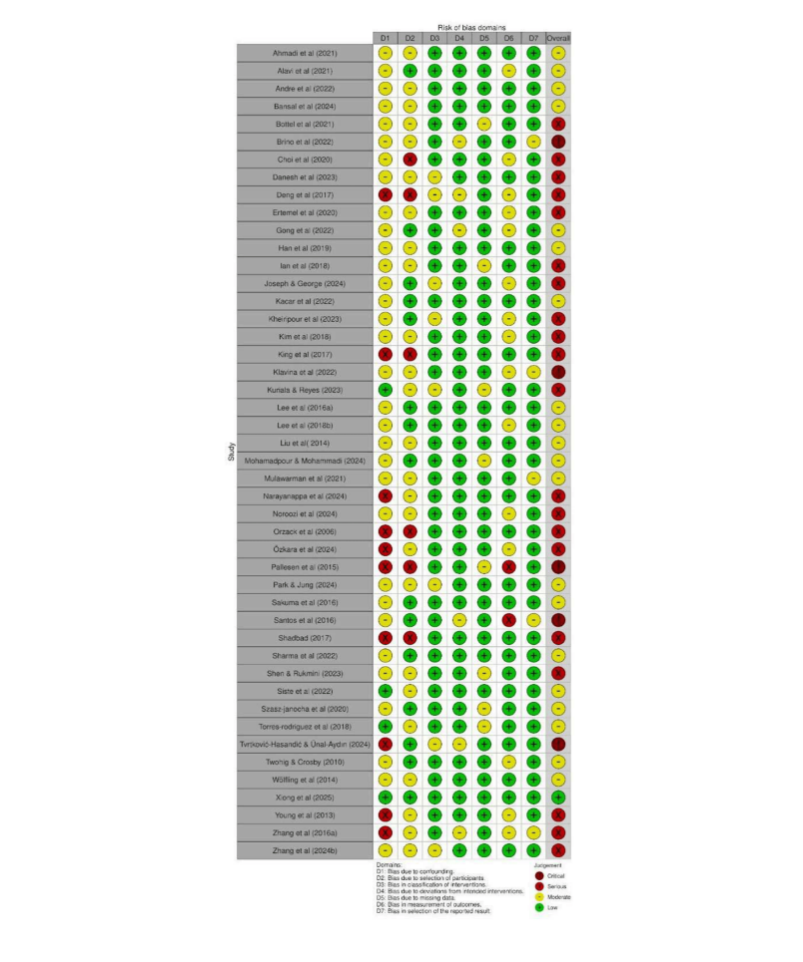
Traffic light figure for nonrandomized and quasi-experimental studies [18,20,25,28,29,33,34,36,39,44-46,49,52,57,59-61,64,65,68,69,73,74,76-78,88-90,93,94,101-103,105,115,119,121,123,130,132,133,136,138].

Based on Cochrane ROBINS-I (for quasi-experimental and pre-post design studies) criteria, 1 (2.1%) study was rated as low risk, 20 (43.4%) as moderate risk, 20 (46%) as serious risk, and 5 (10.8%) as critical risk of bias (Figure 10). The most common sources of bias were related to selective outcome reporting and confounding. Several studies failed to report all predefined outcomes or lacked a prespecified analysis plan, resulting in moderate to serious risk in the “selection of the reported result” domain. These studies additionally demonstrated issues with confounding due to inadequate adjustment for baseline differences. Some studies also showed concerns in participant selection and deviations from intended interventions, largely due to limited methodological reporting. These limitations suggest caution when interpreting effect estimates, especially in studies with elevated risk profiles (Figure 11).

## Discussion

### Principal Findings

This SRMA used a cross-domain frame that treated problematic use of digital technology as a set of related conditions shaped by shared mechanisms but expressed across diverse platforms. It brings together evidence from psychological, digital, behavioral, pharmacological, neuromodulation, physical activity or exercise-based, multicomponent, parental, and VR-based interventions. It included online and offline digital behaviors and spanned age groups and settings. This broad approach allowed direct comparison across intervention types and conditions and helped clarify which approaches show consistent benefit, which remain uncertain, and where the field needs more research. The present work provides a comparative summary of the evidence and provides meta-analytic comparisons and gives the readers a broader global picture of interventions for problematic use of digital technology, while previous literature looked at individual comparisons.

Psychological interventions formed the largest group and showed the most consistent benefit. CBT, ACT, mindfulness, DBT, and skills-based programs reduced symptoms across internet addiction, gaming disorder, smartphone addiction, excessive screen time, pathological gambling, and pornography addiction. Digital and physical activity or exercise-based interventions also produced clear gains, although effects varied across studies. Combined programs that linked behavioral and physical or psychoeducational components showed broad improvements despite their smaller number. Evidence for pharmacological, neuromodulation, VR-based, educational, and parent-focused interventions was limited but showed promise in select contexts.

Meta-analysis showed strong pooled effects for psychological, digital, and exercise-based programs for internet addiction. Psychological and exercise-based interventions were effective for smartphone addiction. Pooled estimates for gaming disorder did not reach significance. High heterogeneity was present in most models.

The current findings are in keeping with the earlier reviews showing that psychological interventions offer benefit in the context of specific addictive behaviors [141]. Previous research noted that CBT reduces maladaptive thoughts and helps users regulate cues and craving [142,143]. Our results support this view. Studies in this review showed that CBT improves control of use and reduces distress across multiple conditions. Mindfulness-based care has shown similar results in earlier work. Li et al [131] found that mindfulness reduces craving and enhances attention control in internet addiction, a pattern mirrored in our findings.

Digital interventions offer wide reach and have gained attention in recent years. Andrews et al [144] reported that web-based CBT and guided self-help improve depressive symptoms when adherence is high. Our findings show a similar trend. Digital programs that used reminders, guidance, or steady practice produced stronger gains than unguided formats. These results suggest that digital care may support scale and access if programs ensure dose, guidance, and engagement. This is of particular relevance in the low- and middle-income countries where uptake of digital interventions for mental (including addictive disorders) is limited [145].

Exercise-based interventions are an emerging area. Weinstein and Lejoyeux [146] proposed that exercise may offset stress and improve self-regulation in those with high digital use. Our review supports this idea. Aerobic activity, yoga, and tai chi reduced craving and improved mood and attention in groups with problematic use of digital technology in the form of the internet and smartphone. These effects were strongest in structured, repeated sessions.

Psychological interventions were found to have a larger effect compared to exercise-based interventions. The findings suggest that strategies focusing on behavioral modifications and physical regulations would benefit from inclusion interventions addressing cognition to mitigate problematic use of digital technology. This aligns with the concept of behavioral addiction, suggesting that problematic use of digital technology involves impairment in impulse control, cognitive distortions, and emotional dysregulation [147].

Pharmacological interventions had mixed support in prior work. The current findings support this view. Antidepressants reduced gaming symptoms in some trials, but many gains were in the context of the mood change rather than change in pattern of use. Stimulants, antipsychotics, and noradrenergic agents had limited effect. Neuromodulation studies showed short-term change in craving and gaming patterns.

Educational and parent-focused interventions showed modest benefit. This is in keeping with the prior research showing that knowledge and education, while important, may not be sufficient on its own to lead to sustained behavior change [148]. In our review, behavior improved only when programs added active tasks, self-monitoring, or steady home involvement. VR-based care has seen early use in exposure therapy for gambling and gaming. VR can reduce some symptoms in the short run, but durability is unclear.

Multicomponent approaches appear promising. Previous reviews argued that problematic digital use is complex and may require programs that target emotion, behavior, and context together [149].

The combination of high heterogeneity, small sample sizes in several intervention groups, and significant Egger tests indicated that results should be interpreted with caution. Selective reporting and methodological variability may have influenced the magnitude of observed effects.

Evidence for problematic use of technology in the context of social media use, pornography watching, gambling, shopping or buying, OTT content watching, and problematic screen time remains limited. The limited research focus on therapeutic interventions for addictive behaviors has been reported previously [150]. Moreover, some digital technology use-related behaviors such as OTT content watching have generated limited research interest [151].

These results suggest that interventions that address linked cognitive, emotional, and behavioral factors in a structured way, with steady practice and clear guidance, produce the most stable gains. These findings also support the use of digital and exercise-based methods to widen reach.

### Strengths and Limitations

This review has several clear strengths. This SRMA adds value beyond earlier work by covering all major forms of problematic use of digital technology in one analysis. Prior reviews focused on single behaviors such as internet addiction [152], gaming disorder [149], or pornography watching [153]. Others limited their scope to one age group, usually adolescents or university students, or to one type of intervention such as CBT [149]. Such narrow scopes helped define early patterns but did not capture how interventions perform across conditions. The concept of generalized and specific internet and digital device use related behaviors continues to be debated in literature [154]. Similarly, recommendations have been made to distinguish essential and nonessential internet use [155]. The current SRMA uses a broad frame that presents integrated synthesis of evidence on problematic use of digital technology. This allowed direct comparison across various behaviors in the context of problematic use of digital. The review covers all major types of interventions, including psychological, educational, digital, pharmacological, exercise or physical activity, VR, neuromodulation, and combined interventions. The review includes both online and offline forms of digital technology use and covers work with adolescents, young adults, and older adults. This wide scope gives a full view of current treatment options. The studies span many regions, which improves the reach and real-world value of the findings. The analysis used standard checks for bias, which adds to the strength of the pooled results. It also points out areas with few studies, such as OTT content watching, shopping or buying, and pornography watching. This synthesis of evidence can help strengthen the earlier recommendations on safe and healthy use of technology [156].

Several limitations affect interpretation of this SRMA. Many studies had small samples and short follow-up. Most relied on self-report measures rather than logs or device data. Intervention dose and fidelity were not always clear, and adherence was rarely tracked in detail. Study quality was mixed. Risk of bias was moderate in many nonrandomized studies, with common issues in outcome reporting, confounding, and documentation of protocol adherence. High heterogeneity reflects variation in intervention format, intensity, and outcome tools. Several nonrandomized studies showed bias due to weak control of baseline traits, unclear steps in the intervention, or gaps in outcome reports. Even among randomized trials, some did not report plans for analysis or checks on how well the intervention was delivered.

It is important to note that the small number of gambling studies included in this SRMA reflects inclusion of only digital or online gambling interventions. A substantial body of literature exists for land-based gambling disorder. However, these studies were beyond the scope of this review [157].

The pooled results also showed high variation across studies. Trials differed in target groups, treatment dose, number of sessions, and the tools used to measure change. This wide spread lowers the strength of the pooled estimates. Tests for small-study effects showed signs of bias in several groups, and this may raise the size of some pooled effects. Some areas, such as social media use, pornography use, gambling, and screen time, had too few studies for pooling. There were no studies on OTT content watching. Most studies came from school or college settings in a few regions. As a result, the findings may not apply to other settings or broader groups.

### Implications of the Findings

The findings of this SRMA show that structured care can reduce problematic use of digital technology. Psychological interventions, such as CBT, ACT, and mindful practice, led to the most steady improvements across conditions. Digital interventions also reduced symptoms when they offered clear steps, brief guidance, or steady practice. This shows that remotely delivered care can reach more people when support is built into the program. Exercise-based work lowered symptoms in several groups and may serve as a useful add-on for people who respond to routine, movement, or changes in mood and focus. Blended models that join psychological interventions with movement or mindful tasks may help by acting on more than one driver of problem use.

The findings also point to gaps in the research that need attention. Studies used many tools, short follow-up, and mixed forms of interventions. A shared set of measures and longer follow-up after treatment would make evidence clearer. Strong evidence is still missing for social media use, pornography watching, gambling, OTT content watching, and screen time. Few studies tested adults in clinic settings, so more work is needed beyond schools and colleges. The findings have specific implications in the context of low- and middle-income countries. Despite well-documented problematic use of digital technology from these countries, the studies exploring the therapeutic interventions remain limited [158-161].

### Future Directions and Conclusions

Future studies should include larger samples, longer follow-up, and standard outcome sets. Clear reporting on intervention dose, adherence, and prespecified plans is needed. Studies should test stepped-care models and evaluate combinations of psychological, digital, exercise, and family-based components. Evidence for adults, clinical populations, and users with high distress is limited and requires expansion.

Across the available evidence, psychological interventions offer the most reliable benefit. Digital and exercise-based interventions also show value and can broaden reach. Combined models may offer added strength by addressing cognitive, emotional, and behavioral drivers together. Pharmacological, neuromodulation, VR-based, and parental interventions show early promise but need further study. Evidence for problematic use of technology in the context of social media use, pornography watching, gambling, shopping or buying, OTT content watching, and problematic screen time remains limited.

Many forms of care can reduce problematic use of digital technology, but sustained change depends on structured tasks, guided support, and continued practice. Programs that follow these principles can help individuals gain control, reduce harm, and restore healthier patterns of technology use.

## Supplementary material

10.2196/89280 Multimedia Appendix 1 Search strings used to search different databases.

10.2196/89280 Multimedia Appendix 2 Summary of the studies included in the systematic review and meta-analysis on therapeutic interventions for the management of problematic use of technology.

10.2196/89280 Checklist 1 PRISMA 2020 Checklist

## Abbreviations

ACT: Acceptance and Commitment Therapy
BGCS: Bergen Internet Addiction Scale
BIS/BAS: behavioral inhibition system or behavioral activation system
CBT: Cognitive Behavioral Therapy
CGI-I: Clinical Global Impression – Improvement Scale
CIAS-R: Chen Internet Addiction Scale - Revised
DBT: Dialectical Behavior Therapy
DLPFC: dorsolateral prefrontal cortex
FSPEP: Fully Supervised Physical Exercise Program
IA: internet addiction
IAT: Internet Addiction Test
*ICD-11*: *International Classification of Diseases, 11th Revision*
IGD: internet gaming disorder
IGDS9-SF: Internet Gaming Disorder Scale-Short Form
IUD: internet use disorder
MBCT: Mindfulness-Based Cognitive Therapy
MeSH: Medical Subject Headings
OTT: over-the-top
PGSI: Problem Gambling Severity Index
PIU: problematic internet use
PRISMA: Preferred Reporting Items for Systematic Reviews and Meta-Analyses
PSPEP: Partly Supervised Physical Exercise Program
RCT: randomized controlled trial
RoB 2: Cochrane Risk of Bias 2.0
ROBINS-I: Risk of Bias in Non-Randomized Studies - of Interventions
SAS-SV: Smartphone Addiction Scale-Short Version
SASA: Smartphone Addiction Scale for Adolescents
SFT: Solution-Focused Therapy
SMD: standardized mean difference
SRMA: systematic review and meta-analysis
tDCS: transcranial direct-current stimulation
VR: virtual reality
XR: extended reality
YDQ: Young’s Diagnostic Questionnaire
YIAS: Young Internet Addiction Scale
YIAT: Young’s Internet Addiction Test
