# Supplementary material for: Therapeutic Interventions Targeted at Problematic Use of Digital Technology: Systematic Review and Meta-Analysis of Evidence
Source: JMIR Ment Health. 2026 May 12;13:e89280. doi: 10.2196/89280 (PMC13167059; doi:10.2196/89280)
Supplement: Multimedia Appendix 1 [file mental-v13-e89280-s001.docx]

| **Table S1. Search strings used to search different database** |
| --- |
| **Pubmed** |
| (("excessive internet*" OR "problematic internet*" OR "pathological internet*" OR "compulsive internet*" OR "internet addict*" OR "online addict*" OR "internet-addicted" OR "addictive internet" OR "internet depend*" OR "internet overuse" OR "internet abuse" OR "internet use" OR "internet usage" OR "video game addiction" OR "video gaming addiction" OR "video game disorder" OR "online game addiction" OR "online gaming addiction" OR "internet gaming disorder" OR "internet game disorder" OR "internet gaming addiction" OR "internet game addiction" OR "computer game addiction" OR "problematic internet gaming" OR "gaming disorder" OR "pathological video gam*" OR "problematic video gam*" OR "problematic online gam*" OR "gaming addiction" OR "video game dependency" OR "excessive gaming" OR "problematic gaming" OR "problematic digital gaming" OR "social network addiction" OR "social networking addiction" OR "pornography addiction" OR "facebook addiction" OR "online shopping addiction" OR "network addiction" OR "digital addiction" OR "social media addiction" OR "problematic social media" OR "digital media addiction" OR "phone addiction" OR "smartphone addiction" OR "mobile phone addiction" OR "cell phone addiction" OR "phone depend*" OR "smartphone depend*" OR "mobile phone depend*" OR "cellular phone depend*" OR "phone overuse" OR "smartphone overuse" OR "cell phone overuse" OR "phone abuse" OR "smartphone abuse" OR "mobile phone abuse" OR "problematic phone" OR "problematic mobile phone" OR "problematic cell phone" OR "problematic cellular phone" OR "problematic smartphone" OR "problem phone" OR "problem mobile phone" OR "problem smartphone" OR "excessive phone" OR "excessive mobile phone" OR "excessive cellular phone" OR "excessive smartphone" OR "smartphone overdependence" OR "smartphone use disorder" OR "smartphone addiction" OR "smartphone overuse" OR "smartphone dependence" OR "online sports" OR "esports" OR "digital media addiction" OR "digital media overuse" OR "digital media dependen*" OR "excessive screen time" OR "screen overuse" OR "Internet Addiction Disorder"[Mesh] OR "Gambling"[Mesh])) AND (((intervention* OR therap* OR treat* OR program* OR strateg* OR reduc* OR train*)) AND ((effectiveness))) |
| **Scopus** |
| ( TITLE-ABS-KEY ( ( "excessive internet*" OR "problematic internet*" OR "pathological internet*" OR "compulsive internet*" OR "internet addict*" OR "online addict*" OR "internet-addicted" OR "addictive internet" OR "internet depend*" OR "internet overuse" OR "internet abuse" OR "internet use" OR "internet usage" OR "video game addiction" OR "video gaming addiction" OR "video game disorder" OR "online game addiction" OR "online gaming addiction" OR "internet gaming disorder" OR "internet game disorder" OR "internet gaming addiction" OR "internet game addiction" OR "computer game addiction" OR "problematic internet gaming" OR "gaming disorder" OR "pathological video gam*" OR "problematic video gam*" OR "problematic online gam*" OR "gaming addiction" OR "video game dependency" OR "excessive gaming" OR "problematic gaming" OR "problematic digital gaming" OR "social network addiction" OR "social networking addiction" OR "pornography addiction" OR "facebook addiction" OR "online shopping addiction" OR "network addiction" OR "digital addiction" OR "social media addiction" OR "problematic social media" OR "digital media addiction" OR "phone addiction" OR "smartphone addiction" OR "mobile phone addiction" OR "cell phone addiction" OR "phone depend*" OR "smartphone depend*" OR "mobile phone depend*" OR "cellular phone depend*" OR "phone overuse" OR "smartphone overuse" OR "cell phone overuse" OR "phone abuse" OR "smartphone abuse" OR "mobile phone abuse" OR "problematic phone" OR "problematic mobile phone" OR "problematic cell phone" OR "problematic cellular phone" OR "problematic smartphone" OR "problem phone" OR "problem mobile phone" OR "problem smartphone" OR "excessive phone" OR "excessive mobile phone" OR "excessive cellular phone" OR "excessive smartphone" OR "smartphone overdependence" OR "smartphone use disorder" OR "smartphone addiction" OR "smartphone overuse" OR "smartphone dependence" OR "online sports" OR "esports" OR "digital media addiction" OR "digital media overuse" OR "digital media dependen*" OR "excessive screen time" OR "screen overuse" OR "Internet Addiction Disorder" OR gambling ) ) AND TITLE-ABS-KEY ( intervention* OR therap* OR treat* OR program* OR strateg* OR reduc* OR train* ) AND TITLE-ABS-KEY ( effectiveness ) ) AND ( LIMIT-TO ( SUBJAREA , "PSYC" ) ) |
| **Embase** |
| ('excessive internet*' OR 'problematic internet*' OR 'pathological internet*' OR 'compulsive internet*' OR 'internet addict*' OR 'online addict*' OR 'internet-addicted' OR 'addictive internet' OR 'internet depend*' OR 'internet overuse'/exp OR 'internet overuse' OR 'internet abuse' OR 'internet use'/exp OR 'internet use' OR 'internet usage'/exp OR 'internet usage' OR 'video game addiction'/exp OR 'video game addiction' OR 'video gaming addiction' OR 'video game disorder' OR 'online game addiction'/exp OR 'online game addiction' OR 'online gaming addiction'/exp OR 'online gaming addiction' OR 'internet gaming disorder'/exp OR 'internet gaming disorder' OR 'internet game disorder' OR 'internet gaming addiction'/exp OR 'internet gaming addiction' OR 'internet game addiction' OR 'computer game addiction'/exp OR 'computer game addiction' OR 'problematic internet gaming'/exp OR 'problematic internet gaming' OR 'gaming disorder'/exp OR 'gaming disorder' OR 'pathological video gam*' OR 'problematic video gam*' OR 'problematic online gam*' OR 'gaming addiction'/exp OR 'gaming addiction' OR 'video game dependency' OR 'excessive gaming'/exp OR 'excessive gaming' OR 'problematic gaming'/exp OR 'problematic gaming' OR 'problematic digital gaming'/exp OR 'problematic digital gaming' OR 'social network addiction' OR 'social networking addiction' OR 'pornography addiction' OR 'facebook addiction' OR 'online shopping addiction' OR 'network addiction' OR 'digital addiction'/exp OR 'digital addiction' OR 'social media addiction'/exp OR 'social media addiction' OR 'problematic social media' OR 'phone addiction' OR 'mobile phone addiction'/exp OR 'mobile phone addiction' OR 'cell phone addiction'/exp OR 'cell phone addiction' OR 'phone depend*' OR 'smartphone depend*' OR 'mobile phone depend*' OR 'cellular phone depend*' OR 'phone overuse' OR 'cell phone overuse'/exp OR 'cell phone overuse' OR 'phone abuse' OR 'smartphone abuse' OR 'mobile phone abuse' OR 'problematic phone' OR 'problematic mobile phone' OR 'problematic cell phone' OR 'problematic cellular phone' OR 'problematic smartphone' OR 'problem phone' OR 'problem mobile phone' OR 'problem smartphone' OR 'excessive phone' OR 'excessive mobile phone' OR 'excessive cellular phone' OR 'excessive smartphone' OR 'smartphone overdependence' OR 'smartphone use disorder' OR 'smartphone addiction'/exp OR 'smartphone addiction' OR 'smartphone overuse'/exp OR 'smartphone overuse' OR 'smartphone dependence' OR 'online sports' OR 'esports' OR 'digital media addiction' OR 'digital media overuse' OR 'digital media dependen*' OR 'excessive screen time' OR 'screen overuse' OR 'internet addiction disorder'/exp OR 'internet addiction disorder' OR 'pathological gambling'/exp OR 'pathological gambling') AND (intervention* OR therap* OR treat* OR program* OR strateg* OR reduc* OR train*) AND effectiveness |
