# Supplementary material for: Therapeutic Interventions Targeted at Problematic Use of Digital Technology: Systematic Review and Meta-Analysis of Evidence
Source: JMIR Ment Health. 2026 May 12;13:e89280. doi: 10.2196/89280 (PMC13167059; doi:10.2196/89280)
Supplement: Multimedia Appendix 2 [file mental-v13-e89280-s002.docx]

| **Table 1. Summary of the studies included in the systematic review and meta analysis on therapeutic interventions for management of problematic use of technology** | | | | | | | | | | | |
| --- | --- | --- | --- | --- | --- | --- | --- | --- | --- | --- | --- |
| **Psychological interventions** | | | | | | | | | | | |
| **S.No** | **Study ID** | **Author, year** | **Country** | **Study design** | **Population** | **Condition** | **Experimental Group** | **Control Group** | **Description of Intervention** | **Outcome Measures** | **Results** |
| **Internet addiction / problematic internet use / internet use disorders** | | | | | | | | | | | |
| **1** | **1** | **Agbaria (2021)** | Israel | RCT | N=160, Mean age experimental group:13.45 (SD = 1.46); control group:13.91 (SD = 1.92), 112 boys, 48 girls, school settings | Internet addiction | n=80 | n=80 | 12 weekly CBT school lessons + coping skills + homework | IAT; Self-Control Scale | IA significantly reduced vs control (M=2.99 vs 3.99; p<.01) |
| **2** | **2** | **Akgül-Gündoğdu & Selçuk-Tosun (2023)** | Turkey | RCT | N=128, Mean age experimental group: 11.34 (SD=1.12) Control: 11.98 (S.D=1.18)64% males, school settings | Problematic internet use | n=64 | n=64 | 12 week SFA programme, parental psychoeducation( 2 times/week): MAgic sphere, miracle questioning | YIAT, NEAS, NEBS, perceived academic success | Significant reduction (35.65→17.07, p<.01). Significant intergroup effect p=.001 |
| **3** | **3** | **Alavi et al. (2021)** | Iran | Quasi-experimental trial | N= 50 internet addicts, 10 females, age range:18-30 years, university based settings | Internet Addiction | n=25 | n=25 | 15-session CBT with psychoeducation, cognitive restructuring, relapse prevention | IAT; WHO-QOL | Significant IA reduction and improved QoL; maintained at 3-month follow-up |
| **4** | **4** | **Anthony 2022** | China | Pilot study | N=6 college students | Problematic Internet Use | n=6 | - | Brief CBT group intervention: maladaptive cognitions, coping, self regulation | Feasibility, acceptability, preliminary therapeutic outcomes | Intervention showed promising therapeutic effects; feasible and well-accepted |
| **5** | **5** | **Bothe et al. (2021)** | Switzerland | RCT | N= 264, internet addicts, 3.8% women, M _age_= 33.2, SD=10.6, web-based settings | Problematic pornography use | n=123 | n=141 | Online CBT + MI for problematic pornography consumption | PPCS; | Significant short-term improvement; negligible difference in actual use time. |
| **6** | **6** | **Brouzos et al. (2024)** | Greece | RCT | N = 47, M _age_=21(SD = 3), 26 females, web based | Internet Addiction | n=24 | n=23 | 2 weeks, 6 online CBT sessions (60min each): self regulation, self monitoring, goal setting | IAT,SRQ | Significant reduction in IAT after intervention (45.5 < 57.3), sustained at 1.5 months FU |
| **7** | **7** | **Danesh (2023)** | Iran | Quasi-experimental | N=72, 9-12 years, school settings | Internet addiction | n=36 | n=36 | 10 parent-child interaction sessions with guided home practice | YIAS; SEQ-C; PSQI | IA reduced (88.14→75.14); sleep improved; self-efficacy increased |
| **8** | **8** | **Du (2010)** | China | RCT | N=56 patients, 12–17 years, gender distribution not mentioned, school-based settings | Internet addiction | n=32 | n=24 | 8 adolescent CBT sessions + parent & teacher psychoeducation | Beard IA; Time-Mgmt; Internet Overuse Self-Rating Scale SDQ | Time-management and emotional outcomes improved more in CBT |
| **9** | **9** | **Ede et al. (2023)** | Nigeria | RCT | N=40 college students aged 18 to 30, 13 males, college settings | Problematic Internet use | n=20 | n=20 | 8-week after-school CBT-based PIU groups | GPIUS-2 | Large reduction vs control (exp M=24.92 vs control M=70.03); maintained at FU |
| **10** | **10** | **El-Ashry et al. (2023)** | Egypt | RCT | N=60, ≥20 age, 30 males, university settings | Problematic internet use | n=30 | n=30 | 7 distress tolerance training sessions (90 min, 4 weeks): crisis survival, reality acceptance, addiction related crises | DTS,PIU,PWBS | PIU↓ (62.93 →52.13), maintained at 3 month FU (52.70). No significant changes in the control group. |
| **11** | **11** | **Kheiripour (2023)** | Iran | NRCT | N=100, Mean age: 15.18 (SD=1.31)  15–18 years, all females, school settings | Internet addiction | BIS-15;BAS15;FFFS-18 Total=48 | BIS-17;BAS-16;FFFS-19 Total=52 | 7-week CBT emotion-regulation training, tailored by BIS/BAS/FFFS profiles | IAT; r-RST, GHQ | IA decreased; BIS subgroup showed largest reductions (p<.01) |
| **12** | **12** | **Kim et al. (2018)** | South Korea | NRCT | N=17, 12–17 years, 7 males, school settings | Problematic internet use | n=17 | No control group | 8 × 120-min CBT sessions: self-monitoring, goal setting, relapse-prevention | IAS; CDI; STAI | IAS declined (59.88→50.71 post; 52.14 at 1-mo FU); mood improved |
| **13** | **13** | **Liu (2015)** | China | Single-arm experimental study | N=92 participants consisting of 46 adolescents with Internet addiction, aged 12–18 years, and 46  their parents, aged 35–46 years, settings not mentioned | Problematic Internet Use | MFGT group (n = 21 families) | Waitlist control (n = 25 families) | 3-day multi-family therapy + 3-month relapse FU | IA diagnosis; parent-child relationship | IA prevalence dropped from 100% to 4.8% post; 11.1% at 3-month FU |
| **14** | **14** | **Liu (2021)** | China | RCT | N=121 , 12-16 years, 91 males, school based | Internet addiction | n=60 | n=61 | 8-week logotherapy-based mindfulness | CIAS; SAS; SDS; coping | IA lower in intervention vs control (58.48 vs 65.93; p<.05) |
| **15** | **15** | **Mathew et al, (2020)** | India | RCT | N= 100 school adolescents | Problematic Internet Use | n=50 | n=50 | 10 week program (2 parental sessions), WLC: intervention after posttest. 3 month FU | PIU scale, sociodemographic data, internet usage pattern, a biopsychosocial functioning tool, and academic performance | Significant PIU reduction, improved bio-psycho-social functioning, academic performance (p<.05), sustained at FU |
| **16** | **16** | **Mulawarman et al. (2021)** | Indonesia and Malaysia | Quasi-experimental (pre-post control group design) | N=56, 14–16 years, 20 males (36%), 36 females (64%), School-based, face-to-face counseling | Problematic internet use | n=28 | n=28 | 6 sessions: reality group counselling (3 weeks, 80 mins/session) | GPIUS2 | Post intervention < Pre intervention scores (p<.05) |
| **17** | **17** | **Narayanappa (2024)** | India | Quasi-experimental (NRCT),single-group pre–post design | N=144, 14–16 years age, 103 male, school settings | Problematic internet use | n=144 | No control group | 5-day school psychoeducation (awareness, craving control, relaxation, mindful use) | IAT-20 | IAT reduced at 1- and 2-month FU (44.75→34.73→28.84; F=38.085, p<.001) |
| **18** | **18** | **Pu et al. (2023)** | China | RCT | N=26, control: 19.73 ± 1.34 years, treatment:19.50 ± 1.22 years, M: F= 11:15, University settings | Internet addiction | n=14 | n=12 | 5-week SFBT program(1 session/week): goal setting, scaling, miracle imagination | CIAS-R,Future Time Perspective sub-scale of the Revised Chinese Zimbardo Time Perspective Inventory | Significant CIAS-R reduction post intervention, sustained 1-month and 6 month FU |
| **19** | **19** | **Quinones et al. (2019)** | United Kingdom | RCT | N=994 participants, 388 females, M_age_ of mindfulness, MR, and control groups were 39, 40, 41, respectively, web-based settings | Problematic Internet Use | Mindfulness (n =343), Relaxation (n =301) | Wait-list control (n =350) | 2-week app-based mindfulness vs relaxation vs control (10 min/day) | CIU | Mindfulness group improved most; significant vs control |
| **20** | **20** | **Santos et al. (2016)** | Brazil | Open-label, uncontrolled pre-post intervention trial | N=39, Mean age= 28.56 ± 5.93 years  19–42 years age, 12 males, Institute of Psychiatry, UFRJ, Brazil | Internet addiction | n=39 | No control group | 6-week CBT psychoeducation combining cognitive restructuring, coping strategies, social-skills training, and alternative rewarding activities | SOGS; PGSI; craving scale | Gambling severity reduced (p<.05); cravings declined; better coping and self-regulation reported |
| **21** | **21** | **Shadbad (2017)** | Iran | Quasi-experimental study | N=24 adolescent school girls, Mean age=18 years, school settings | Internet Use Disorders | n=12 | n=12 | 8-session MBCT (breathing, PMR, body scan, thought awareness) | Y-IAT; GHQ-28 | IA decreased and general health improved (p<.001) |
| **22** | **22** | **Szasz-janocha et al. (2020)** | Germany | NRCT | N=54, 9–19 years age, Mean age=13.48 ± 1.72, 16.7% females, Outpatient, university and addiction counseling centers | Internet use disorders | n=54 | No control group | 4 week PROTECT program, CBT based group sessions (100 minute): internet use risk factors | CIUS, CSAS-SR/PR, DIKJ, SIAS, PHOKI, SDQ-SR/PR, APROF, FEEL-KJ, SSL, SWE | Significant 12-month IUD symptom reduction (CIUS: 23.49→14.14; CSAS-SR: 16.75→9.75), sustained at 12 month FU |
| **23** | **23** | **Tadpatrikar et al. (2024)** | India | RCT | N= 30, Group A (Yoga + Psychotherapy): 22.9 years (±6.5),Group B (Psychotherapy only): 22.5 years (±5.7)15–40 years, 22 males, clinic at a hospital | Excessive internet use | n=15 | n=15 | 12 week study, 2 arms: psychotherapy+yoga;psychotherapy+booster session | s-IAT, SAS-SV, IGDS9-SF, K6 | Psychotherapy+yoga significantly reduced internet use (F = 5.61, P = 0.02), smartphone use (F = 4.76, P = 0.03) as compared to psychotherapy alone. |
| **24** | **24** | **Wölfling et al. (2014)** | Germany | single-arm experimental study | N=37, all males,Mean age=26.1 ± 6.6 years over 16 years of age, clinics | Internet Addiction | n=37 (26 completed treatment) | No control group (single arm) | STICA: 15 group + 8 individual sessions | AICA; hours; self-efficacy | Large reductions (p<.001; d=1.31–1.45); daily use ↓ to ~2.6 h |
| **25** | **25** | **Wölfling et al. (2019)** | Germany | multicenter RCT | N=143, Mean age= 26.2 ± 7.8 years Men aged 17 to 55 years, hospital settings | Internet and Computer game addiction | n=72 | n=71 | 15-week CBT (STICA) vs waitlist | AICA-S; AICA-C; hours | STICA group showed significantly lower scores and less usage |
| **26** | **26** | **Yang et al. (2022)** | China | RCT | N= 43, Mean age: intervention=19.5 ± 0.8, control=19.9 ± 0.8, 13 males, college settings | Internet Addiction | n=21 | n=22 | 5 weekend psychoeducation sessions focusing on coping & meaning | YIAT; SOC; MSPSS; GPS | Lower YIAT and procrastination; improved sense of coherence |
| **27** | **27** | **Young et al. (2013)** | United States | Single arm pre-post | N=128 | Internet Addiction | - | - | Single arm pre-post 12 CBT-IA sessions (weekly): harm reduction, abstinence; 1,3, and 6 month FU | IADQ, outcome checklist | Significant decrease in IADQ scores (4.63→2.42, p<.05), sustained at FU |
| **28** | **28** | **Zeidi et al. (2020)** | Iran | RCT | N=80, Mean age= 21.7 ± 2.21 years, 40 males, 40 females, university settings | Internet addiction | n=40 | n=40 | 10-week GCBT program: cognitive restructuring, behaviour training, emotional regulation, relapse prevention | YIAT,BSCS,CIUS,GSE | GCBT significantly reduced YIAT scores (F=183.302, p <0.001) and compulsive use (F=163.359, p <0.001) |
| **29** | **29** | **Zhang et al. (2020)** | China | RCT | N=18, Mean age= 20.11 (1.45), 6 males, university settings | Internet addiction | n=9 | n=9 | 5 week group counselling sessions (2.5h/week): FU at 5 weeks, 6 months | CIAS-R, IA progress, satisfaction, SCL-90 | Consistent reduction in CIAS-R (58.78→49.33), sustained at 6 month FU (45.78) |
| **30** | **30** | **Zhao & Pan (2022)** | China | RCT | N=100,Mean age= Control: 15.16 ± 2.18; Intervention: 15.25 ± 2.12, 68 males, Guilin Mental Health Center | Internet addiction | n=50 | n=50 | 12-week psychosocial mutual-help group vs standard withdrawal | CIAS-R; self-efficacy; QoL | CIAS-R 75.26→46.89 (intervention) vs 75.32→63.26 (control) |
| **31** | **31** | **Zhong (2011)** | China | RCT | N= 57, Mean age= E-17.88 (±3); C-18.32 (±2) , 50 males, Inpatient medical setting: Addiction Medical Treatment Center of the Beijing Military Zone General Hospital | Internet addiction | n=28 | n=29 | 14-week family-based CBT group | OCS; FAD; PSSS; Mach IV | Larger IA decrease vs control at post, 1- and 3-month FU |
| **Internet gaming disorder / problematic gaming / problematic online gaming/ gaming disorder** | | | | | | | | | | | |
| **S.No** | **Study ID** | **Author, year** | **Country** | **Study design** | **Population** | **Condition** | **Experimental Group** | **Control Group** | **Description of Intervention** | **Outcome Measures** | **Results** |
| **1** | **32** | **Alexandra Torres-Rodriguez (2018)** | Spain | quasi-experimental study | N=34 participants,Mean age=PIPATIC = 15.19 ± 1.9; CBT = 14.73 ± 1.58 , all males, mental health settings | Internet Gaming disorder | n=17 | n=17 | PIPATIC: 22 weekly sessions over 6 months, including psychoeducation, CBT, intrapersonal and family modules, relapse-prevention & lifestyle planning | IGD-T | Both groups improved, but PIPATIC showed greater symptom reduction (M=32.19 vs 51.93; p=.001) |
| **2** | **33** | **Deng et al. (2017)** | China | NRCT | N=63, Mean age: CBI+= 21.86 (SD = 1.90);CBI−=22.05 (SD = 1.81), settings not mentioned, gender distribution not mentioned | Problematic gaming | n=44 | n=19 | 6-week Craving Behavioral Intervention (CBI+): weekly 2.5–3h mindfulness-based craving control | POGUS; VAS craving; BDI; BAI; AUDIT-C; FTND; BIS-11;Psy-needs | Strong group×time interaction (F=9.08, p<.001); sustained IGD reduction |
| **3** | **34** | **Han et al. (2019)** | South Korea | Quasi-experimental study | N=205 patients, Mean age= CBT: 25.9 ± 5.1,Supportive: 26.5 ± 5.5in hospital settings | Internet Gaming Disorder | n=101 | n=104 | 14 CBT group sessions vs supportive therapy; focus on stress-coping, emotional regulation, identity and family dynamics | YIAS | CBT > control (F=11.76, p=.001) |
| **4** | **35** | **He et al. (2021)** | China | RCT | N=48 subjects 18–22 years old, 9 males, university settings | Internet gaming disorder | n=24 | n=24 | 4-day cognitive bias-modification: avoid gaming cues, approach neutral stimuli | IGD severity; craving; BDI; BAI; BIS-11 | Lower IGD scores (33.79 vs 37.92) and craving vs control; reaction-time bias reduced |
| **5** | **36** | **Ji et al (2023)** | China | RCT | N=77 , Mean age= 16.36 (0.93),14-21 years, Most of them were males (88.3%), school settings | Problematic internet gaming | n=38 | n=39 | 8-week ICBT program, 90 minute/week: game cognition, behaviour, | CIAS, OGPNQ, C-RIGCS, PHQ-9, GAD-7 | ICBT significantly reduced gaming time, internal motivations and GD symptoms (LMM: strong main and group x time effects) |
| **6** | **37** | **Joseph & George (2024)** | India | NRCT | N=100 ppts, age and gender details not mentioned, school settings | Problematic video gaming | n=50 | n=50 | 5-day structured program (18 youth + 2 parent sessions) | GAS; SIAS; RSES; wellbeing | Decreased gaming but NS (p=.054); slight gains but no short-term significance |
| **7** | **38** | **Jung & Park (2025)** | South Korea | RCT | N=47, exercise group Mean age of 12.26  years (SD =1.25, range =10–14 years), consisting of 16  middle school students (all males) and 7 elementary school  students (4 males, 3 females), art therapy group Mean age of 11.96 years (SD =1.46, range =9–14 years),  including 15 middle school students (11 males, 4 females)  and 9 elementary school students (8 males, 1 female) | Internet gaming disorder | n=23 | n=24 | 8-day residential program: art therapy vs exercise camp | K-scale; K-YSR | Both reduced addiction scores (F=20.417, p<.001); no between-group difference |
| **8** | **39** | **Kaya et al. (2023)** | Turkey | RCT | N=44, Mean age= 15.63± 0.49-IG; 15.59± 0.50-CG  16 males,, school settings | Problematic internet use | n=22 | n=22 | 4-month SFA program (7 sessions, 45-60min each) + 6 month FU: miracle questioning,exception finding, parental involvement | IAT, PCIAS, PSQI, HEC | Significantly reduced IAT (57.0→13.0), PCIAS (34.0→13.5, p<0.001), sustained at 6 months FU |
| **9** | **40** | **King et al. (2017)** | Australia | single arm experimental study | N= 24 participants (20 males, 4 females) aged 18 years to 36 years, web-based settings | Internet Gaming Disorder | n=9 | n=15 | 84-hour voluntary abstinence from gaming + daily monitoring & 7-/28-day FU | Hours; maladaptive cognitions; IGD symptoms | 75% showed clinically meaningful IGD improvement at 28-day FU |
| **10** | **41** | **Kuriala & Reyes (2023)** | Phillipines | NRCT | N=30, 16–19 years age, university settings | Internet gaming disorder | n=15 | n=15 | 8-week Acceptance & Cognitive-Restructuring Program (ACRIP): cognitive restructuring + mindfulness + emotion work | IGDS9-SF; PWB | IGD dropped (37.8→18.47); PWB ↑; highly significant (t=15.98, p=.001) |
| **11** | **42** | **Li et al. (2017a)** | United States | RCT | N= 30, 24 males, 5 females, 1 neither gender, university settings | Internet Gaming Disorder | n=15 | n=15 | MORE: 8 weekly 2-h mindfulness-based gaming treatment vs supportive group | DSM-5 IGD; craving; maladaptive cognitions | MORE > SG on all outcomes; benefits sustained to 3-month FU |
| **12** | **43** | **Ni (2024)** | China | RCT | N=64, Mean age= "MM = 20.3 ± 1.9 years  PMR = 20.2 ± 1.5 years  "Adults aged 18 years, 31 males, hospital settings | Internet gaming disorder | n=32 | n=32 | 8-session Mindfulness Meditation vs Progressive Muscle Relaxation | IAT; QGU; fMRI cues | MM > PMR: IGD (−3.6 vs −1.1); cravings ↓ (t=−8.66; η²=.30; p<.001) |
| **13** | **44** | **Nielsen et al. (2021)** | Switzerland | Open-label parallel arm RCT | N= 42, 97.6% males, mean age =14.9 ± 2.0 age range: 12-19 years, hospital-based settings at Phoenix centre hospital | Internet gaming disorder | n=12 | n=30 | MDFT (2×/week) vs FTAU (1×/week), 6-month family-based program | IGD severity (adolescent & parent reports) | MDFT > FTAU at 6 & 12 months; F(1,30)=8.56, p<.01, η²=.22 |
| **14** | **45** | **Nuske et al. (2025)** | Australia | RCT | N= 337; 77 females (23 %) and 248 males (74 %). M_age_ =25 years (SD = 3.2), online based video and audio training | Internet gaming disorder | Mindfulness = 113, Relaxation = 109 | n=115 | Detached mindfulness vs relaxation meditation vs neutral task | Gaming urge (VAS) | Time effect strong (p<.001); relaxation < control (p=.026); mindfulness NS vs control |
| **15** | **46** | **Pallesen et al. (2015)** | Norway | Pre post study | N=12, 12-20 years, all male | Video game addiction | n=12 | No control group | 13 session eclectic therapy: CBT, SF, family and motivational techniques | GASA, PVP, GCI | Patient: moderate improvement, non-significant. Mothers:significant reduction gaming behaviours. Therapist: 6/12 patients show marked improvement. |
| **16** | **47** | **Park 2016a** | Korea | RCT | N=36, All Mean age= CBT 24.2 ± 3.2 ; VRT 23.6 ± 2.7 ; Casual gamers 23.3 ± 2.9 above 18 age, all males, university medical center settings | Online game addiction | CBT=12; VRT=12 | n=12 | CBT and VRT sessions on gaming motives, stress management, family relationships | YIAS, FC and ALFF via fMRI | Significant reduction, CBT > VRT |
| **17** | **48** | **Park & Jung (2024)** | Korea | NRCT | N=27 adolescents ,11 and 15 years old | Internet gaming disorder | n=27 | No control | 8-day Equine-assisted learning program (15 sessions) | K-YSR; Korean IGD scale | IGD dropped 76.52→57.35; partial relapse at 1-month FU (64.74) |
| **18** | **49** | **Sakuma et al. (2016)** | Japan | Single-arm experimental study | N= 10 patients with IGD, all males, M_age_ = 16.2 years, Deaddiction centre settings | Internet Gaming Disorder | n=10 | No control | 9-day tech-free residential camp with CBT, lectures & counseling | SOCRATES; gaming time | Gaming time ↓ at 3-month FU; motivation & self-efficacy ↑ |
| **19** | **50** | **Sharma et al. (2022)** | India | Single-arm experimental study | N=40 participants M_age_ 20.25, SD = 5.39, 39 males and 1 female, hospital settings | Internet gaming disorder | Single group (n = 33 completers) | No control | 10-session motivational + CBT intervention | IAT; IGD-20; WHOQOL-BREF | Pre-post IAT (52.88→42.87; t=5.10, p=.000); IGD-20 also ↓ (t=6.94, p=.000) |
| **20** | **51** | **Wei (2025)** | China | RCT | N= 55, 18-30 years, Mean age="IA N= 19.61 ± 1.19  IA+EX = 19.39 ± 1.73  " 43.3% females in AAT group and 40% in the Sham-AAT group, hospital lab settings | Internet Gaming Disorder | n=30 | n=25 | ApBM, Sham-AAT, pretest-posttest | IAT; Game Craving Questionnaire (Chinese Version, ApBM Assessment, fMRI data analysis logs | Significant reduction in ApBM group (12.112 ± 6.118 to −6.186 ± 4.155.) No significant change in Sham-AAT group. |
| **21** | **52** | **Xu et al. (2024)** | China | RCT | N=61, 30 females, M _age_= 20.35 (1.89) to 20.23 (1.53), hospital settings | Internet gaming disorder | n=31 | n=30 | 4-week mindfulness vs PMR (8 sessions; twice/week) | Craving; IAT; DSM-5; fMRI | Strong interaction (F=22.558, p<.001); MM ↓ craving & IGD more than PMR |
| **22** | **53** | **Zhang et al. (2016)** | China | NRCT | N=59, M _age_:IGDs=21.95 ± 1.84; HCs=22.89 ± 2.23, all males, OP Based settings | Internet gaming disorder | n=40 | n=19 | 6-session CBI with mindfulness + cue-reactivity task | CIAS; BAI, BDI; Cigarette and alcohol use and the Fagerstrom Test for Nicotine Dependence; Alcohol Use Disorders Identification Test; gaming duration | Large reductions in CIAS (82.09→60.26; p<.001) & craving; control improved less |
| **Smartphone addiction / problematic smartphone use / mobile phone dependence** | | | | | | | | | | | |
| **S.No** | **Study ID** | **Author, year** | **Country** | **Study design** | **Population** | **Condition** | **Experimental Group** | **Control Group** | **Description of Intervention** | **Outcome Measures** | **Results** |
| **1** | **54** | **Choi et al. (2020)** | Korea | Quasi-experimental study | N=49, 8 males, M_age_= 16 years, school settings | Smartphone addiction | n=24 | n=25 | 12-week classroom program: guided meditation, emotional reflection, mindfulness toward smartphone use | Smartphone Addiction Self-Report Scale; Self-Control; Stress | Significant reductions in smartphone addiction, instant and long-term satisfaction (all p<.001) |
| **2** | **55** | **Hu (2025)** | China | RCT | N=88, 10-16 age, 50% males, school settings | Problematic smartphone use | PREIP-IG = 30, CBT-CG = 28 | W-CG = 30 | PREIP: 8-week individual emotional-processing intervention vs CBT vs waitlist | MPPUS-10; MPAI; CLS; PSSS; AERQ; PANAS; SWLS | PREIP > CBT for sustained reduction (34.30→26.07→22.30); CBT relapsed slightly; waitlist unchanged |
| **3** | **56** | **Lan et al. (2018)** | China | quasi-experimental study | N=70 participants, M_age_ =21.3 ± 1.3 years, males=47.6% of the sample, college settings | Smartphone addiction | n=41 | n=29 | 8 week GMCI+CBT and mindfulness: cognitive restructuring, relapse prevention, mindfulness | MPIAS, smartphone use time | Significant reductions in both MPIAS and smartphone use time: initially after intervention; more reductions in control group at 20 week FU |
| **4** | **57** | **Lee et al. (2016)** | South Korea | Single-arm experimental. study | N= 46, 13 males, M_age_=13.22 ± 0.89, school settings | Smartphone addiction | n=46 | No control | 2-week HDJ-S CBT-based daily smartphone diary + parent discussion + self-evaluation | Korean Smartphone Addiction Proneness Scale; Motive Scale; Parental Concerns | Significant addiction reduction (mean=33.59±4.59, t=5.87, p<.001); parental concern ↑ (p<.05) |
| **5** | **58** | **Liu (2024)** | China | RCT | N = 44 , 17–22 years, mindfulness = 17–20 years control, 13 males, university settings | Problematic mobile phone use | n=22 | n=22 | 40-minute body-scan mindfulness recording vs neutral news control | MPATS; FMI; C-MLQ | Mindfulness > control: ↑ mindfulness (p=.013), ↑ meaning in life (p<.001), ↓ phone addiction (p=.008) |
| **6** | **59** | **Liu (2024)** | China | RCT | N= 94, 12 to 18 years of age, 44 boys and 50 girls, school settings | Problematic smartphone use | n=45 | n=49 | 8-session adolescent MBCT: body scan, sitting meditation, attention & emotion regulation, homework | DASS-21; PSQI; SAS-SV; CAMM | Lower PSU, depression, sleep problems; higher mindfulness at post and 2-month FU vs control |
| **7** | **60** | **Ochs & Sauer (2021)** | Switzerland | RCT | N=97, 18 to 29 years, 81.4% females, naturalistic field settings experiment | Problematic smartphone use | n1=37, n2=37 | n3=28 | Behavioral UI nudges: greyscale display or relocating apps; no restriction of function | Screen-time; pickups; MPPUS; PANAS; SWLS; UEQ-S | PSU increased across all groups (F(1,94)=5.29, p<.05); no difference between intervention types (NS) |
| **8** | **61** | **Song et al. (2016)** | South Korea | 3 arm RCT | N= 119 adolescents and adults, all males, M_age_= bupropion SR: 20.0±3.62, Escitalopram- 19.8±4.2, observation group- 19.6±4.0, hospital settings | Internet gaming disorder | Bupropion (n = 44), Escitalopram (n = 42) | Observation group (n = 33); no medication | Bupropion (150mg/day → 300mg/day) vs escitalopram (10mg/day →20mg/day) | YIAS,CHI-S, BDI, ARS, BIS/BAS | Significant improvement in both groups, bupropion >escitalopram (CGI-S, YIAS, ARS, BIS) |
| **9** | **62** | **Tanşu (2023)** | Turkey | RCT | N=104, 17- 34 years, 36 males, university settings | Smartphone addiction | n=52 | n=52 | 6-session online psychoeducation: addiction awareness, neurobiology, CBT basics, time management, emotional regulation, coping/assertiveness | SAS-SV | Smartphone addiction significantly lower at post-test, 3- & 6-month follow-ups (p<.05) |
| **10** | **63** | **Wang & Chen (2023)** | China | RCT | N=56, 28 males, M_age_ =19.1 ± 0.5 years, community settings | Mobile phone addiction | n=28 | n=28 | 8-week mindfulness-based mental-health education (attention control, emotional regulation, time mgmt, self-acceptance) + daily home practice | MPAI; GHQ-12; CAOT | Large MPAI reduction (57.32→44.68, p<.001) vs small non-sig change in control; all subscales improved (p<.001) |
| **11** | **64** | **Xiong et al.(2025)** | China | Quasi-experimental | N=60, no age details, 16 males, university settings | Problematic smartphone use | n=30 | n=30 | 8-week MBCT + Metaphor Therapy for problematic social media use | Problematic Social Media Use Scale; hrs/day | PSMU ↓ from 4.00±0.36→3.16±0.56, maintained at 3-mo FU; control unchanged (F=0.936, p=.398) |
| **12** | **65** | **Zhang (2024)** | China | Quasi-experimental | N=232, no age details, 130 boys, community based | Mobile phone addiction | n=119 | n=113 | 12-week HAPA-based weekly counseling: motivation building → action planning + behavior change | SAS-SV; GSES; CASLSS | Addiction ↓ 46.45→36.37 (t=11.23, p=.001); control unchanged; significant between-group difference (t=–12.27, p=.001) |
| **Problematic pornography watching** | | | | | | | | | | | |
| **S.No** | **Study ID** | **Author, year** | **Country** | **Study design** | **Population** | **Condition** | **Experimental Group** | **Control Group** | **Description of Intervention** | **Outcome Measures** | **Results** |
| **1** | **66** | **Crosby (2016)** | United States | RCT | N=28, M_age_ =29.3 years (SD = 11.4), all males, clinic/ University settings, | Problematic online pornography viewing | n=14 | n=14 | 12-week individual Acceptance & Commitment Therapy (ACT) for problematic pornography use; weekly 1-hour manualized sessions plus homework and therapist feedback | DPVQ, QOLS, SCS, CBOSB | Significant reduction in pornography use (F(1,25)=6.42, p=.018); 93% reduction vs 21% in controls; 54% complete cessation post-treatment; 86% reduction maintained at 3-month follow-up |
| **2** | **67** | **Orzack (2006)** | United States | Single arm experimental study | N= 35, M_age_= 44.5 years, all males, addiction study centre settings | Problematic Internet-enabled sexual behavior | 5 closed groups (avg. 6–8 members) | No control | 16-week closed group program using CBT + psychodynamic + motivational interviewing; weekly structured sessions; homework and peer-support phone calls to improve coping and social engagement | OTIS, BASIS-32, BDI | Emotional and depressive symptoms improved (BASIS-32 Δ=1.98; BDI Δ=2.63), but reduction in problematic computer use was **not significant** (OTIS Δ=1.5; p=0.135) |
| **3** | **68** | **Twohig & Crosby (2010)** | United States | NRCT | N=6, aged 21–39, all males, university research lab settings | Problematic Internet pornography viewing | n=6 | No control group | Eight weekly ACT sessions (90 min) focusing on acceptance, cognitive defusion, mindfulness, values, and relapse prevention; homework and metaphoric exercises | Self-monitoring logs, QOLS, OCI, AAQ, TAF, TCQ | ~86% reduction in pornography viewing time; most participants reduced to near-zero use; gains largely sustained at follow-up |
| **Problematic gambling/ gambling disorder** | | | | | | | | | | | |
| **S.No** | **Study ID** | **Author, year** | **Country** | **Study design** | **Population** | **Condition** | **Experimental Group** | **Control Group** | **Description of Intervention** | **Outcome Measures** | **Results** |
| **1** | **69** | **Andre (2022)a** | Sweden | single arm experimental study | N=9 children and adolescents, 12- 17 years age, eight (89%) male, hospital settings | Problem gambling | Single group (n = 9) | No control | CBT (7 sessions, 45 minutes)) Motivation enhancement, relapse prevention for DG and PG | GASA | DG symptom reduction,no change in PG. |
| **2** | **70** | **Houghton & Moss (2024)** | United Kingdom | RCT | N= 281, 57 females, 18-64 years age, message based | Problematic online sports betting | n1= 95 , n2= 92 | n=88 | Two-week exposure to tailored responsible-gambling messages on Twitter (harm-minimisation, self-control tips, normative feedback) | Gambling frequency; Sports Betting Behaviour Questionnaire,Readiness to Change Questionnaire;amount wagered; self-reported harm | Small reductions observed across both groups; no statistically significant differences between messaging exposure and comparison group |
| **3** | **71** | **Luquiens (2016)** | France | RCT | N= 1122, 34.7 years mean age, Most of them were male, (1033/1122, 92.07%), web based | Problematic online gambling | n=858 | n=264 | Online programme comprising personalized normative feedback, self-help CBT modules, and therapist-guided CBT, delivered over 6 weeks | PGSI; self-efficacy; gambling frequency; expenditure | All arms showed decreases from baseline; no significant differences between conditions at post-test |
| **4** | **72** | **Smith (2015)** | Australia | Two-group randomised, parallel design | N= 87, M_age_= exposure group: 45.50 (12.04), Cognitive group: 47.45 (13.88), females= 44, Gambling Therapy/ mental health care centre | Problematic gambling | CT (n = 44), ET (n = 43) | No passive control; both groups received active treatment | Twelve weekly individual sessions comparing Cognitive Therapy (cognitive restructuring of gambling beliefs) with Exposure Therapy (graduated cue exposure with urge management) | Victorian Gambling Screen (VGS); Gambling-Related Cognitions Scale (GRCS); Kessler-10; Work & Social Adjustment Scale (WSAS) | Both treatments reduced gambling-related cognitions and distress; clinical improvements seen across time with no large differences between conditions |
| **Social media addiction/ problematic social media use** | | | | | | | | | | | |
| **S.No** | **Study ID** | **Author, year** | **Country** | **Study design** | **Population** | **Condition** | **Experimental Group** | **Control Group** | **Description of Intervention** | **Outcome Measures** | **Results** |
| **1** | **73** | **Manwong (2018)** | Thailand | Cluster RCT | N=245, 12–15 years of age, 116 males, school settings | Social media addiction | n=125 | n=120 | 4-week school-based CBT psychoeducation plus media-literacy and time-management training, delivered in small groups | IGDS-S; self-control; usage log | IGD scores significantly decreased (p<.001); self-control increased; reduced hours/day at 1-month follow-up |
| **2** | **74** | **Yang et al (2023)** | China | RCT | N= 66, 17–24 years, 18 men, university settings | Social network addiction | n=33 | n=33 | Mindfulness integration (into life (formal +informal practices) | SMD, SAS, SDS, PSS | Significant effects on addiction level (F= 39.39, p <.00) |
| **Problematic screen time/ excessive screen time** | | | | | | | | | | | |
| **S.No** | **Study ID** | **Author, year** | **Country** | **Study design** | **Population** | **Condition** | **Experimental Group** | **Control Group** | **Description of Intervention** | **Outcome Measures** | **Results** |
| **1** | **75** | **Conroy et al. (2017)** | United States | RCT | N=204, 21–60 years, 76% female, Remote (self-monitoring via handheld device) | Excessive screen time | n=204 | No control | 3-week CBT-based group program integrating success building and self efficacy cognitive restructuring, coping-skills training, and relapse-prevention; self monitored screen time, digitally reported. | MVPA, screen time log, Marlowe-Crowne Social Desirability Scale | Increased daily physical activity, reductions in screen time, sustained over 20-week FU. |

| **Educational Interventions** | | | | | | | | | | | |
| --- | --- | --- | --- | --- | --- | --- | --- | --- | --- | --- | --- |
| **Internet gaming disorder / problematic gaming / problematic online gaming/ gaming disorder** | | | | | | | | | | | |
| **S.No** | **Study ID** | **Author, year** | **Country** | **Study design** | **Population** | **Condition** | **Experimental Group** | **Control Group** | **Description of Intervention** | **Outcome Measures** | **Results** |
| **1** | **76** | **Chung (2023)** | South Korea | RCT | N=126 students, M_age_ =12.1 years, 66 boys, school settings | Problematic internet gaming | Coding group (n = 58) | Literacy group (n = 68) | 4 weeks, 2 parallel programs 8 sessions each on game coding and literacy | YIAS,K-SPIN, PHQ-9, Self efficacy, K-ARS | Significant reductions on YIAS in both groups, game coding>literacy. No significant change in internet use time in both groups. |
| **2** | **77** | **Männikkö et al. (2022)** | Finland | One group Pre test/Post-test | N=22 aged between 18 and 28 (M = 23.05, SD = 3.02) | Gaming Disorder | N=22 | **-** | 3 month,10 session psychoeducational intervention: early prevention and awareness | \Gaming problems severity, average  gaming time per day, quality of life, mental well-being | Slight reductions in gaming time (weekdays: *d* = 0.13, *p* = .545; weekends: *d* = 0.08, *p* = .714). Severity (*d* = 0.17, *p* = .411); non-significant. |
| **3** | **78** | **Zamanian (2020)** | Iran | RCT | N=64 | Computer game addiction | N=32 | N=32 | 8-session educational program (TPB-based) + parent workshop | Gaming dependency questionnaire; TPB constructs | Scores dropped 54.4→49.1 post-test (p≤.001); small rebound at FU |
| **Smartphone Addiction / Problematic Smartphone Use / Mobile Phone Dependence** | | | | | | | | | | | |
| **S.No** | **Study ID** | **Author, year** | **Country** | **Study design** | **Population** | **Condition** | **Experimental Group** | **Control Group** | **Description of Intervention** | **Outcome Measures** | **Results** |
| **1** | **79** | **Ertemel et al. (2020)** | Turkey | Single-arm experimental. study | N=305 students (48.2% girls and 51.8% boys), M _age_ =14.57 (SD = 0.74), school settings | Smartphone addiction | n=305 | No control | One-day educational programme, 8 modules: problematic smartphone use, track screen time, improve digital environment | SASA | Significant reduction in PSU (16.19, p<0.01) after intervention. |
| **2** | **80** | **Noroozi et al. (2024)** | Iran | NRCT | N=72, Only females, 4-6th grade primary school M_age_ intervention=11.19 ± 1.09, M_age_ control =10.75 ± 1.2 | Mobile phone addiction | n=36 | n=36 | Transtheoretical Model–based counseling + weekly motivational messages and self-monitoring tasks for 8 weeks | Decisional Balance; MPA; self-efficacy | Significant reduction in problematic phone use and higher readiness for change (p<.001); self-efficacy increased |
| **3** | **81** | **Shen & Rukmini (2023)** | Indonesia | NRCT | N=54, age and gender details not mentioned, university settings | Smartphone addiction | Print= 23;Social=27 | No control group | One-month educational programme, 2 sessions, 2 groups (printed leaflets, line based infographics.) | SAS, ISEL, RSES | Printed Media<Infographics (103.26 <123.70, p =0.000), Printed media showed substantial reduction in SAS. |

| **Digital Interventions** | | | | | | | | | | | |
| --- | --- | --- | --- | --- | --- | --- | --- | --- | --- | --- | --- |
| **Internet addiction / problematic internet use / internet use disorders** | | | | | | | | | | | |
| **S.No** | **Study ID** | **Author, year** | **Country** | **Study design** | **Population** | **Condition** | **Experimental Group** | **Control Group** | **Description of Intervention** | **Outcome Measures** | **Results** |
| **1** | **82** | **Ahmadi (2021)** | Iran | NRCT | N= 268, M_age_ = 21 years, all females, Shahid Beheshti University of Medical Sciences, Tehran, Iran | Internet addiction | n=134 | n=134 | 8-week educational intervention: booklets and SMS, based on HBM | IAT, HBM components, IA behaviour and frequency. | Slight decrease in IA behaviour, but not significant (p=0.11) |
| **2** | **83** | **Bansal et al. (2024)** | India | NRCT | N= 40, Both genders, M_age_= 24, 18-30 years | Internet Addiction | n=20 | n=20 | 2-day online meditation training (20min) guided mediation (20min) for next 8 weeks, online meditation sessions twice a week. | YIAT, WHOQOL-Bref, MOCA | Significant time and group effects observed for YIAT post-intervention (p <0.001). |
| **3** | **84** | **Bernstein et al. (2023)** | Germany | RCT | N= 130 M_age_ =28.45 (SD=10.59), women: 65 (50%) | Internet Addiction | n=65 | n=65 | 4-6 week CBT Intervention: guidance from eCoach, 1-2 session/week. | IAT, CIUS, AQoL-8, PHQ-9, GAD-7, CSQ-8, user satisfaction. | Participants in the IG achieved significantly lower IUD symptom severity on the IAT than the WCG (p<0.001) |
| **4** | **85** | **Besser et al. (2022)** | Germany | RCT | N= 36,M _age_=33.51 (SD = 12.06), female representation of 55.7 % | Internet Addiction | n=20 | n=16 | MI: 1 in-person + 3 phone calls + personalized feedback letter | CIUS | Intervention vs control, not statistically significant (p=.216) |
| **5** | **86** | **Bottel 2021** | Germany | Single-arm experimental study | N= 140, 119 males with IUD, > 18 years, telemedicine settings | Internet Use Disorders | n=73 | n=67 | Telemedicine consultations using La-Well Systems software; 2 sessions: interviews, behavioral tasks, and motivational strategies. | iSOCRATES (motivation), s-IAT (symptom severity), Internet use hours/day | Significant increase in motivation to change, substantial IYD symptom reduction (2hrs/day) |
| **6** | **87** | **Brino et al 2022** | United States | Pre post study | N=28, 12 to 17 years age, M _age_=14.2 years, 71.4% female, pediatric OP behavioral health clinic | Problematic internet use | n=28 | No control group | 5-week telehealth intervention for PIUL education, peer support, therapy. | PhQ-ADS, GPIUS_2, Screen time | PIU↓ (86.5 →70.3, therapeutic impact (t = 12.07, p < 0.001) |
| **7** | **88** | **Chashmi et al. (2024)** | Iran | RCT | N=36 high-school and university students aged 18–40  years; 25 females and 11 males | Internet Addiction | n=18 | n=18 | 20 sessions eWMT, adaptive dual sensory tasks, placebo completed non-adaptive task. | IAT-SF, BART, CERQ | PIU ↓ significant change in IAT scores only in the intervention group. |
| **8** | **89** | **Dieris-Hirche (2023)** | Germany | RCT | N=180, M _age_: Exp= 32.1(13.0),Cont= 31.5(11.4), 136 male, university medical centers settings | Internet use disorders | n=89 | n=91 | 4 week telemedicine OMPRIS intervention: motivational interviewing, CBT, ACT | AICA-S; PHQ-933, GAD-7; iSOCRATES, GSE, Life satisfaction | OMPRIS significantly reduced AICA-S scores versus control (12.1→ 6.8; p < 0.0001). |
| **9** | **90** | **Fu et al. (2025)** | China | RCT | N=60 participant, 33 males and 27 females, 18 years or older, hospital lab settings | Internet gaming disorder | n=30 | n=30 | Cognitive-bias modification targeting gaming cues; approach-avoidance retraining | approach bias scores, IAT, DSM5, Craving, fMRI | ApBM significantly reduced IAT DSM-5, craving, and approach bias. No significant changes in sham-ApBM. |
| **10** | **91** | **Ma (2024)** | China | RCT | N= 145, M _age_ =22.85, 78.6% women, online social media platform | Internet addiction | n=64 | n=81 | 10-day photography and reflection intervention: | IAT-12, DASS-21The Positive and Negative Emotion Scale, The Shapiro Control Inventory, The Meaning in Life Questionnaire,The short version of the Multidimensional Scale of  Perceived Social Support | Intervention reduced IAT scores more than WLC (F=5.51, p=0.007, ηp²=0.04); larger effect in intervention group. |
| **11** | **92** | **Mohamadpour & Mohammadi (2024)** | Iran | Quasi-experimental (Pre-test-Post-test with control group) | N=40, 12-18 years age, all females, online based | Internet addiction | n=20 | n=20 | 8-week integrative therapy program: emotion regulation, reduced maladaptive coping. | YIAT, IARS, BPD for children and adolescents | Intervention reduced IA (F=117.02, p<0.001), sustained at FU (F=122.40, p<0.001). |
| **12** | **93** | **Nieman (2023)** | Germany | Multicentric RCT | N=169 , M_age_ =31.9 (SD 12.1) years, 75.1% male, 1.8% diverse, 23.1% females, web based | Problematic Internet use | n=81 | n=88 | 4-week intervention, 8 remote sessions: motivation to change behaviour, reduction in media addiction. | AICA-S;EQ-5D-5L QLYs | Improvement in media addiction symptoms. |
| **13** | **94** | **Shahrajabian (2023)** | Iran | RCT | N=36, 18–40 years, 25 females, 11 males, Remote/home-based digital training | Problematic internet use | n=18 | n=18 | 20 session eWMT: affective dual task, placebo involved non-affective shape tasks. | IAT, Go/No-Go, CPT, Wechsler Digit Span (FDS/BDS) | Significant reduction in PIU (68.17 → 40.22), sustained effects at FU  Minimal change in placebo group (67.05 → 66.67) |
| **14** | **95** | **Siste et al. (2022)** | Indonesia | Quasi-experimental trial | N= 40, affiliated universities and hospitals during covid 1st wave,Exp,M_age_: 19.4 ± 1.27, control M_age_: 20.3 ± 1.94; 17 males and 23 females | Internet Addiction | n=20 | n=20 | 8-session DBT-IA vs 4-session CBT-IA | IAT; URICA; duration | Both reduced IA; no significant difference between arms |
| **15** | **96** | **Su et al. (2010)** | China | 4 armed RCT | N=65l, 45 women and 20 men, University settings | Internet addiction | LE (n = 17), NE (n = 16), NI (n = 16) | Waitlist control (n = 16) | CBT modules (HOSC system): self awareness, behavioural changes across four groups (LE,NE, NI, Control) | Online hours/week, YDQ, Satisfaction, legitimate use ratio | HOSC, YDQsignificantly reduced in LE, NE; NI shows similar results. |
| **Internet gaming disorder / problematic gaming / problematic online gaming/ gaming disorder** | | | | | | | | | | | |
| **S.No** | **Study ID** | **Author, year** | **Country** | **Study design** | **Population** | **Condition** | **Experimental Group** | **Control Group** | **Description of Intervention** | **Outcome Measures** | **Results** |
| **1** | **97** | **Ding et al. (2023)** | China | RCT 2x3 design, active intervention | N=90 | Gaming disorder | N=49 (ACT group), N=41 (engaged ACT group) | - | 2 online self help ACT intervention (10 sessions, 2 weeks): Full ACT + Engaged ACT program, 1 month FU | Assessment 3 times (pre-, mid- and post-program): GD, psychological flexibility, daily gaming hours, weekly gaming days,psychological distress, | Reductions in GD and weekly gaming frequency, psychological flexibility ↑. Engaged ACT >Full ACT: daily gaming hours. NO change in psychological distress. |
| **2** | **98** | **Andre (2023)a** | Sweden | RCT | N=102, 13–18 years age, 75 males, Outpatient CAP clinics in Region Skåne | Internet gaming disorder | n=47 | n=55 | 5–7 individual relapse-prevention CBT sessions (45 min each), delivered in-person or via video by trained clinicians (psychologists, social worker, psychiatrist). Components included goal-setting, identifying high-risk situations, early warning signs, and reinforcing new activity schedules, with homework each session. Control group received treatment-as-usual (counseling, ADHD medication, antidepressants, referral, evaluation, or waitlist/discharge) | Game Addiction Scale for Adolescents (GASA) | Both groups’ GASA scores declined significantly over time; RP showed stronger improvement. Significant time × condition interaction (p<.001) and medium effect size (d=0.77). Follow-up scores lower in RP vs TAU; within-group reductions significant in both arms |
| **3** | **99** | **Kacar & Ayaz-Alkaya (2022)** | Turkey | Quasi experimental study | N=44 school children, between the ages of 8 and 11, 17 girls, school settings | Internet addiction | n=20 | n=22 | Children participated in Turkish games over 8 weeks, 3 times a week (60min/session) | The Family–Child Internet Addiction scale, the Social Skills Assessment Scale, the Social Skills Scale, and the Perceived Stress Scale in Children (8–11 years) | Significant reduction in daily/weekly internet use (p<0.05) |
| **Smartphone addiction / problematic smartphone use / mobile phone dependence** | | | | | | | | | | | |
| **S.No** | **Study ID** | **Author, year** | **Country** | **Study design** | **Population** | **Condition** | **Experimental Group** | **Control Group** | **Description of Intervention** | **Outcome Measures** | **Results** |
| **1** | **100** | **Keller (2021)** | Germany | RCT | N=232, 18–60 years, 88% female, 10% male, 1% diverse, Remote (app-based intervention) | Problematic smartphone use | n=114 | n=118 | 20-day intervention, 5 sequential 4-day modules: mindfulness, impulse control. | MPPUS, iOS screen time, self efficacy and planning scales | Both the intervention and control conditions led to notable reductions in problematic smartphone use and overall daily |
| **2** | **101** | **Zhang (2025)** | China | RCT | N=101., M_age_=20.82, 21 males and 83 females, online settings | Problematic smartphone use | n=57 | n=44 | 30-day brief online mindfulness: breath-counting, breath-watching, body scanning (10 min/day) | SAS-SV; MSNA; MGA; MIAA; MSVA; MAAS | Experimental group showed significant reductions across addiction subscales and higher mindfulness |
| **Social media addiction/ problematic social media use** | | | | | | | | | | | |
| **S No** | **Study ID** | **Author, year** | **Country** | **Study design** | **Population** | **Condition** | **Experimental Group** | **Control Group** | **Description of Intervention** | **Outcome Measures** | **Results** |
| **1** | **102** | **Yaman & Yilmaz (2023)** | Turkey | RCT | N= 110, M _age_ =21.6 ± 1.5, 67.6% of the participants were female, online sessions | Social media addiction | n=51 | n=54 | Twelve-session online RAM-based intervention to reduce social media addiction by promoting awareness, encouraging behavioral change, and fostering healthier lifestyle habits | BSMAS-Social Media Addiction Scale;  HLBS-II, Healthy Lifestyle Behaviours Scale. | No BSMAS difference (p>0.05); intervention improved HLBS-II and REI significantly (p<0.05) |
| **Problematic screen time/ excessive screen time** | | | | | | | | | | | |
| **S.No** | **Study ID** | **Author, year** | **Country** | **Study design** | **Population** | **Condition** | **Experimental Group** | **Control Group** | **Description of Intervention** | **Outcome Measures** | **Results** |
| **1** | **103** | **Raj et al. (2023)** | Malaysia | ClusterRCT | N=360 dyads, M_age_: Mother- Exp=34.1 (4.3); Cont=33.4 (4.7), school based settings | Excessive screen time | n=180 | n=180 | Four-week mother-focused programme using YouTube animation and WhatsApp problem-solving guidance; weekly videos plus facilitated behaviour-change reminders | Child screen-time logs; parent-reported behaviour tracking | Children’s daily screen time reduced and maintained; parents reported improved consistency in household rules and monitoring |

| **Pharmacological interventions** | | | | | | | | | | | |
| --- | --- | --- | --- | --- | --- | --- | --- | --- | --- | --- | --- |
| **Internet addiction / problematic internet use / internet use disorders** | | | | | | | | | | | |
|  |  |  | **Country** | **Study design** | **Population** | **Condition** | **Experimental Group** | **Control Group** |  |  |  |
| **1** | **104** | **Dell'Osso, 2008** | United States | 10 week open label, followed by 9 week double blind place controlled trial | N=19;  Responders: 14 | Excessive Internet Use | 7 | 7 | 10 week open label escitalopram trial (10-20mg/day), 9 week double-blind placebo- controlled phase: impulsive-compulsive IUD | CGI-I, non-essential IU hours | Significant decrease in IU (36.8→16.5 hrs/week; *p* = .002), CGI-I responders =64.7%. Placebo vs. escitalopram: no significant difference. |
| **Internet gaming disorder / problematic gaming / problematic online gaming/ gaming disorder** | | | | | | | | | | | |
| **S.No** | **Study ID** | **Author, year** | **Country** | **Study design** | **Population** | **Condition** | **Experimental Group** | **Control Group** | **Description of Intervention** | **Outcome Measures** | **Results** |
| **1** | **105** | **Nam et al. (2017)** | South Korea | RCT | N=30, M_age_:Bupropion= 22.9 ± 1.9; Escitalopram= 23.9 ± 1.6, , Psychiatric outpatient clinics | Internet gaming disorder | Bupropion + psychoeducation (n = 15);Escitalopram + psychoeducation (n = 15) | No control group | Bupropion (150mg/day) and escitalopram (10mg/day) titrated to 300mg/day and 20mg/day respectively in the first week. Stable daily dosage was maintained from week 2-12. | YIAS< BDI, K-ARS, BIS-BAS, fMRI resting-state readings | Both treatments reduced IGD symptoms (p<0.01); bupropion showed greater improvement, though group difference nonsignificant (p=0.13)  . |
| **2** | **106** | **Park et al. (2016)** | Korea | RCT | N=36, All above 18 age, all males, university medical center settings | Online game addiction | CBT=12; VRT=12 | n=12 | MPH (10mg/day→40mg/day), ATM (10mg/day→60mg/day) during the first 2 weeks | YIAS, CDI, ADHD- RS, BIS, BAS | No significant changes in YIAS and BIS/BAS scores. MPH>ATM scores at 3 month FU |
| **Problematic gambling/ gambling disorder** | | | | | | | | | | | |
| **S.No** | **Study ID** | **Author, year** | **Country** | **Study design** | **Population** | **Condition** | **Experimental Group** | **Control Group** | **Description of Intervention** | **Outcome Measures** | **Results** |
| **1** | **107** | **Fong et al. (2008)** | United States | RCT; double-blind | N= 21, 18- 65 years, 10 females, Gambling center | Problematic video poker gambling | n=9 | n=12 | 7week trial, placebo vs olanzapine (2.5g-10mg): craving scales, gambling logs | BGCS, DGS, CGI-PG, BPRS, BDI, HAM-A, HAM-D, BIS | Reduction in gambling urges in both groups, with decreased gambling time and higher craving in the treatment group. |

| **Physical/ Exercise based Intervention** | | | | | | | | | | | |
| --- | --- | --- | --- | --- | --- | --- | --- | --- | --- | --- | --- |
| **Internet addiction / problematic internet use / internet use disorders** | | | | | | | | | | | |
| **S.No** | **Study ID** | **Author, year** | **Country** | **Study design** | **Population** | **Condition** | **Experimental Group** | **Control Group** | **Description of Intervention** | **Outcome Measures** | **Results** |
| **1** | **108** | **Klavina et al. (2022)** | Latvia | Controlled parallel-group quasi-experimental study | N=27, 11–14 years, 10 males, School-based and home-based physical activity | Problematic internet use | PSPEP (n = 14), FSPEP (n = 13) | No control group | 15 week intervention comparing PSPEP and FSPEP over 48 sessions conducted 3 times/week (60-90 minutes) | PRIUSS (PIU), BOT-2 (motor), VTS (cognitive) | No significant PIU changes observed in PSPEP or FSPEP groups (p>0.05), minimal effect of physical activity interventions on reducing PIU |
| **2** | **109** | **Zhang et al (2023)** | China | RCT | N=93, M_age_= 20.23, M:F= 12: 9, University settings | Internet addiction | exercise group:n=31, tai chai group:n=31 | n=31 | 8 week programme involving sports conventional exercise, tai chi, and control (3 times/week), standard intensity | PSQI, SDS, SAS, FS-14, IAT | Exercise significantly reduced IAT score Vs control (p=0.004), tai chi showed no significant difference. |
| **Smartphone addiction / problematic smartphone use / mobile phone dependence** | | | | | | | | | | | |
| **S.No** | **Study ID** | **Author, year** | **Country** | **Study design** | **Population** | **Condition** | **Experimental Group** | **Control Group** | **Description of Intervention** | **Outcome Measures** | **Results** |
| **1** | **110** | **Lu et al (2020)** | China | RCT | N=95, M _age_=19.23 years, 68 males, university settings | Problematic smartphone use | ME-31,CBT-30 | n=34 | 12-week ME-Qigong vs CBT vs control (2×/week, 90-min) | MPAI-C; SRAS; ULS-8; PSS-14 | ME-Qigong > CBT > control in reducing PSU (F(2,58)=13.604, p<.001); ME significantly lower final scores |
| **2** | **111** | **Xiao et al. (2021)** | China | RCT | N=96, M_age_: Baduanjin = 19.21 ± 1.02; Basketball = 18.95 ± 0.89; Control = 19.71 ± 1.77, 71 males, university settings | Problematic smartphone use | n=62 | n=34 | 12-week, 36 sessions: structured physical activity (3 times/week). Parallely Baduanjin group practiced mind-body guidance. | MPAI-chinese version, SRAS, ULS-8, FIS, CPSS) | Both interventions produced statistically significant reductions in PSU (p<0.01). Less improvement in the control group. |
| **3** | **112** | **Yang et al. (2022)** | China | RCT | N=60, 18 to 22 years, 30 males, university settings | Mobile phone dependency | n=30 | n=30 | Exercise group completed treadmill sessions at 45–68% heart rate reserve (HRR), control group listened to music. | RPE, VAS, ,Physical Activity Rating Scale-3,Mobile Phone Addiction Tendency Scale | Significant moderate reduction in mobile phone craving in exercise group ( 3.77 ± 1.36) compared to the control group (VAS score: 6.12 ± 1.39) |
| **4** | **113** | **Zhang et al. (2024)** | China | Quasi-experimental | N=232, no age details, 130 boys, community based | Mobile phone addiction | n=119 | n=113 | 3 groups: tai chi, aerobic exercise, control group (8 week program, 24 sessions) | ,PMPU, ADS< SAS, RSES,GSES,PSQI, FS-14 | Significant reduction in PMPU by aerobic, tai chi groups. No significant difference between groups. |
| **Problematic screen time/ excessive screen time** | | | | | | | | | | | |
| **S.No** | **Study ID** | **Author, year** | **Country** | **Study design** | **Population** | **Condition** | **Experimental Group** | **Control Group** | **Description of Intervention** | **Outcome Measures** | **Results** |
| **1** | **114** | **Özkara et al. (2024)** | Turkey | Quasi experimental design | N=176, 57.4% of the participants were aged 21 years and younger and 42.6% of the participants were aged 22 years and older, 102 males, university settings | Excessive screen time | n=85 | n=91 | Recreational exercise programme, 3 times a week over several weeks including basketball, Futsal and Volleyball. | SVT, LBS | Significant reduction in screen time (1.55h→1.14h) post intervention. Control group showed slight increase1.73 → 1.78 hours |

| **Neuromodulation-based intervention** | | | | | | | | | | | |
| --- | --- | --- | --- | --- | --- | --- | --- | --- | --- | --- | --- |
| **Internet gaming disorder / problematic gaming / problematic online gaming/ gaming disorder** | | | | | | | | | | | |
| **S.No** | **Study ID** | **Author, year** | **Country** | **Study design** | **Population** | **Condition** | **Experimental Group** | **Control Group** | **Description of Intervention** | **Outcome Measures** | **Results** |
| **1** | **115** | **Jeong et al. (2020)** | South Korea | RCT-single-blind, sham-controlled trial | N=26, M_age_ Active tDCS=22.2 ± 1.7 yrs;Sham tDCS=23.2 ± 1.6 yrs, 26 males, clinical and imaging centre settings | Internet gaming disorder | n=13 | n=13 | 4 week, 12 session active/sham tDCS intervention (3 times/week for 30 mins each) | IAT, BSCS, BIS/BAS, weekly gaming hours, FDG-PET scans for rCMRglu | Significant reduction in active tDCS group in gaming time (16.1 to 9.4 hours, p = .005, d = 0.75). No significant changes in the control group. |
| **2** | **116** | **Lee et al. (2018)** | South Korea | Pre-post study | N=25, M_age_Gamers=21.3 ± 1.4 years; M_age_ Non-gamers= 28.8 ± 7.5 years, Gamers: 8 males, 7 females; Non-gamers: 6 males, 4 females hospital settings | Internet gaming disorder | n=15 | n=10 | 12 tDCS sessions (2 mA, 30 min) targeting bilateral DLPFC, scheduled 3 times a week. | IAT, BSCS, BDI-II, weekly gaming hours, FDG-PET scans for cerebral glucose metabolism | Significant reduction in problematic gaming behaviour. IAT scores dropped from 37.5  to 24.9 (p < .001), and weekly gaming hours decreased from 16.8 to 10.3 (p = .02). |
| **3** | **117** | **Wu et al. (2021)** | China | RCT | N=38, all males, 18–25 years old, Cognitive neurosciece laboratory settings | Internet gaming disorder | n=19 | n=19 | Crossover design: active (1.5 mA) and sham tDCS over right dlPFC, cravings assessed pre- and during-stimulation. | Background and cue induced craving, inhibitory control (reaction time) | Active tDCS reduced background craving (p<0.001), sham and cue induced craving showed no significant changes. |

| **Combined/Multi-component based intervention** | | | | | | | | | | | |
| --- | --- | --- | --- | --- | --- | --- | --- | --- | --- | --- | --- |
| **Internet addiction / problematic internet use / internet use disorders** | | | | | | | | | | | |
| **S.No** | **Study ID** | **Author, year** | **Country** | **Study design** | **Population** | **Condition** | **Experimental Group** | **Control Group** | **Description of Intervention** | **Outcome Measures** | **Results** |
| **1** | **118** | **Gong et al. (2022)** | China | NRCT | N=118 ,no age and gender details, Community-based school settings, with structured sessions delivered in designated counseling and exercise spaces | Internet addiction | n=60 | n=58 | 8 week dual modality program: narrative group counselling, therapy sessions, Pilates exercises. Once a week (45-60mins) | Self-rating Young’s Diagnostic Questionnaire for IA,- GHQ-12 (PANAS),SCL-90: for screening only  g | Intervention significantly reduced IA (Δ = 1.61 ± 0.58, p < 0.001), between-group difference robust (t=6.422, p<0.001). |
| **2** | **119** | **Li et al. (2017b)** | China | RCT | N=120, 79 males, mean age for EA grp- 4.7±1.9, comprehensive Rx grp- 4.7±2.1, Psycho- intervention- 4.2±2.0 | Internet addiction | CT=40,EA=40, PI=40 | No control group | EA was administered using EA device (), PI component followed a cognitive behavioural framework. In CT group, participants received both EA (10 sessions/course) and PI (5 sessions) | IA Scores, SCL 90 self report scale for mental health, Latency and Amplitude of P50 | Significant reductions in IAD scores (p<0.05). the combined EA plus PI group exhibited the greatest improvement, with scores dropping from 75 ± 8 to 40 ± 11. |
| **Internet gaming disorder / problematic gaming / problematic online gaming/ gaming disorder** | | | | | | | | | | | |
| **S.No** | **Study ID** | **Author, year** | **Country** | **Study design** | **Population** | **Condition** | **Experimental Group** | **Control Group** | **Description of Intervention** | **Outcome Measures** | **Results** |
| **1** | **120** | **Han & Renshaw (2012)** | South Korea | RCT | N=50, 13-45 age, all male, Chung Ang University Medical Center, Seoul | Excessive online game play (EOP) | n=25 | n=25 | 8-week RCT: bupropion SR (150→300 mg/day) vs placebo + weekly psychoeducation on healthy Internet use, addiction harms, and coping strategies | YIAS; BDI; CGI-S; weekly gaming hours | Significant reductions in IGD severity and gaming time in the bupropion group (p<.01). YIAS: 71.5→45.2 vs 68.5→59.2 in placebo. Gaming hours: 47.3→21.1 vs 44.3→29.8. Improvements sustained at 4-week follow-up |
| **Smartphone addiction / problematic smartphone use / mobile phone dependence** | | | | | | | | | | | |
| **S.No** | **Study ID** | **Author, year** | **Country** | **Study design** | **Population** | **Condition** | **Experimental Group** | **Control Group** | **Description of Intervention** | **Outcome Measures** | **Results** |
| **1** | **121** | **Bong et al. (2021)** | South Korea | RCT | N=155, 10–16 years, 68 males, school settings | Smartphone and internet addiction | n=67 | n=71 | 8-week program combining weekly group CBT (via “Smart Me” Home-based Daily Journal with weekly written tasks) and structured music therapy (rhythm work, emotional expression, craving-focused lyric/percussion exercises, collaborative songwriting) | SAPS; YIAT; SAIC; TAIC; BDI-II; CASS(S); BIS-11; RSES | Significant reductions in smartphone and Internet addiction (p<.001). SAPS: 32.75→26.48; YIAT: 46.79→32.16. Improvements also observed in emotional symptoms |
| **2** | **122** | **Li et al. (2025)** | China | RCT | N= 66, 18-21 age, all male, college settings | Mobile phone addiction | n=33 | n=33 | 8-week Mindfulness-Based Tai Chi Chuan (3 sessions/week): wuji stance with affirmations, Yang-style Tai Chi, relaxation with music, breathing and seated meditation | MAAS; MPAI; executive-function test | Significant reduction in mobile-phone addiction: MPAI 46.09 vs 56.55 in control (Δ=−10.46; p=.016). Improved mindfulness and executive function |

| **Intervention with parents** | | | | | | | | | | | |
| --- | --- | --- | --- | --- | --- | --- | --- | --- | --- | --- | --- |
| **Internet addiction / problematic internet use / internet use disorders** | | | | | | | | | | | |
| **S.No** | **Study ID** | **Author, year** | **Country** | **Study design** | **Population** | **Condition** | **Experimental Group** | **Control Group** | **Description of Intervention** | **Outcome Measures** | **Results** |
| **1** | **123** | **Sela et al, 2025** | Israel | RCT | 157 | Problematic Internet Use | n=92 | n=65 | 3 PVC sessions parent training program: active and restrictive mediation strategies, parental regulation of children’s online behaviour | Parent+child self report questionnaires;smartphone monitoring- baselines, post intervention, 8 week FU | PVC group reported reduced helplessness, fewer conflicts,  improved child functioning; reduced problematic internet use scores for children |
| **Problematic screen time/ excessive screen time** | | | | | | | | | | | |
| **S.No** | **Study ID** | **Author, year** | **Country** | **Study design** | **Population** | **Condition** | **Experimental Group** | **Control Group** | **Description of Intervention** | **Outcome Measures** | **Results** |
| **1** | **124** | **Xie et al. (2025)** | China | 2 arm RCT study | N=336 | Excessive screen time | N=168 | N=168 | 2 arm, cluster-randomised, WLC design: multicomponent intervention to reduce screen time | Screen time assessment:daily/weekly/weekends; entertainment and academic. | Significantly reduced daily+weekly screen time (p<0.005); entertainment screen time (β = −22.29 to −14.40, *p* < 0.05). No significant effects for parenting practices/physical activity |

| **Virtual Reality–Based Interventions** | | | | | | | | | | | |
| --- | --- | --- | --- | --- | --- | --- | --- | --- | --- | --- | --- |
| **Internet gaming disorder / problematic gaming / problematic online gaming/ gaming disorder** | | | | | | | | | | | |
| **S.No** | **Study ID** | **Author, year** | **Country** | **Study design** | **Population** | **Condition** | **Experimental Group** | **Control Group** | **Description of Intervention** | **Outcome Measures** | **Results** |
| **1** | **125** | **Tvrtković-Hasandić (2024)** | Bosnia and Herzegovina | NRCT | N=27, M _age_:RGU= 21.5 ± 2.76; IGD= 20.33 ± 1.12; HC= 22.63 ± 2.50, University ,psychology lab settings | Problematic internet gaming | IGD group (n = 9) | Recreational gamers (n = 10), non-gamers (n = 8) | 4 week, 20 minute weekly mindfulness sessions : promoting focus awareness, and emotional acceptance via XRHealth Portal. | IGDT-10, PSS_10, PANAS-SF, IAT | Significant decrease in IGD symptoms (2.22→0.89) and weekend gaming time (3.56h→2.89h). No significant change in weekday gaming time. |
| a the study Andre (2022) also included participants with disordered gaming. However, these were also included in the final publication based on this study and was reported as part of Andre (2023). The results for disordered gaming for Andre (2022) have not been presented to avoid duplication. | | | | | | | | | | | |
